# Horizontally acquired regulatory gene activates ancestral regulatory system to promote *Salmonella* virulence

**DOI:** 10.1093/nar/gkaa813

**Published:** 2020-10-12

**Authors:** Jeongjoon Choi, Eduardo A Groisman

**Affiliations:** Department of Microbial Pathogenesis, Yale School of Medicine, 295 Congress Avenue, New Haven, CT 06536, USA; Department of Microbial Pathogenesis, Yale School of Medicine, 295 Congress Avenue, New Haven, CT 06536, USA; Yale Microbial Sciences Institute, P.O. Box 27389, West Haven, CT 06516, USA

## Abstract

Horizontally acquired genes are typically regulated by ancestral regulators. This regulation enables expression of horizontally acquired genes to be coordinated with that of preexisting genes. Here, we report a singular example of the opposite regulation: a horizontally acquired gene that controls an ancestral regulator, thereby promoting bacterial virulence. We establish that the horizontally acquired regulatory gene *ssrB* is necessary to activate the ancestral regulatory system PhoP/PhoQ of *Salmonella enterica* serovar Typhimurium (*S*. Typhimurium) in mildly acidic pH, which *S*. Typhimurium experiences inside macrophages. SsrB promotes *phoP* transcription by binding upstream of the *phoP* promoter. SsrB also increases *ugtL* transcription by binding to the *ugtL* promoter region, where it overcomes gene silencing by the heat-stable nucleoid structuring protein H-NS, enhancing virulence. The largely non-pathogenic species *S. bongori* failed to activate PhoP/PhoQ in mildly acidic pH because it lacks both the *ssrB* gene and the SsrB binding site in the target promoter. Low Mg^2+^ activated PhoP/PhoQ in both *S. bongori* and *ssrB*-lacking *S*. Typhimurium, indicating that the SsrB requirement for PhoP/PhoQ activation is signal-dependent. By controlling the ancestral genome, horizontally acquired genes are responsible for more crucial abilities, including virulence, than currently thought.

## INTRODUCTION

**Figure 1.**
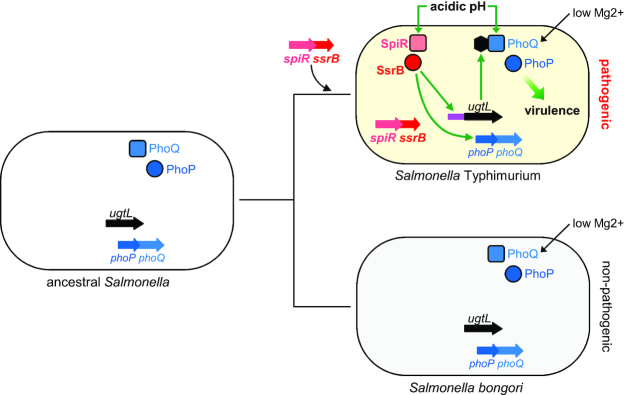
The horizontally acquired regulatory *ssrB* gene promotes *S*. Typhimurium virulence by activating the ancestral PhoP/PhoQ system in mildly acidic pH. The ancestral organism that gave rise to *S*. Typhimurium and *S. bongori* harbored the ancestral *phoP* and *phoQ* genes specifying the PhoP/PhoQ regulatory system and the horizontally acquired *ugtL* gene. *S*. Typhimurium acquired the *spiR* and *ssrB* genes by horizontal gene transfer, which, together with the evolution of an SsrB binding site in the *ugtL* promoter (purple), enables activation of PhoP/PhoQ in mildly acidic pH (by promoting transcription of *ugtL* and *phoP* genes) and virulence. The non-pathogenic species *S. bongori* lacks the *spiR* and *ssrB* genes and the SsrB binding site in the *ugtL* promoter, and is unable to activate PhoP/PhoQ in mildly acidic pH. Low Mg^2+^ activates the PhoP/PhoQ system in both *S*. Typhimurium and *S. bongori*. The SsrB protein is a direct transcriptional activator of the *phoP* gene. Thus, the evolved function of the ancestral regulatory circuit by transferred foreign gene(s) distinguishes organisms’ behavior.

Horizontal gene transfer plays a key role in microbial evolution because it can readily endow a recipient organism with new abilities (1–4). Expression of horizontally acquired genes can be independent of the ancestral genome but is most often controlled by ancestral regulators (5–10). This control enables bacteria to coordinate expression of newly acquired genes with that of preexisting genes, thereby avoiding potential negative fitness effects resulting from their uncoordinated expression (1,8–10). Here, we provide a singular example of the antithesis: a horizontally acquired regulatory gene that controls an ancestral regulatory system required for bacterial virulence (Figure 1). This control allows bacteria to expand the environments in which a regulator operates.

The genus *Salmonella* consists of two species: *S. enterica*, which includes >2500 serovars, many of which responsible for disease conditions in humans (11), and *S. bongori*, which is largely non-pathogenic (12). *S. enterica* serovar Typhimurium (*S*. Typhimurium) requires a horizontally acquired gene cluster known as *Salmonella* pathogenicity island 2 (SPI-2) to survive inside macrophages and to cause a typhoid fever-like disease in mice (13,14). SPI-2 specifies several virulence determinants, including a regulatory system—designated SsrB/SpiR (15,16)—that controls transcription of SPI-2 genes and of other horizontally acquired genes scattered around the *S*. Typhimurium genome (16–19). SsrB is a DNA-binding transcriptional regulator activated by the sensor SpiR (sometimes referred to as SsrA) (15,16). Transcription of the horizontally acquired *ssrB* and *spiR* genes is regulated by several ancestral regulators, including SlyA (20), OmpR (6), and PhoP (7).

The PhoP and PhoQ proteins form a two-component regulatory system that governs virulence in *S*. Typhimurium (21–23) (Figure 1). The sensor PhoQ responds to inducing signals by promoting the phosphorylated state of the DNA binding regulator PhoP (PhoP-P), the active form of the PhoP protein (24). The UgtL protein increases the fraction of PhoP-P by advancing PhoQ autophosphorylation (25). PhoQ activation by mildly acidic pH is critical for *S*. Typhimurium virulence because inhibition of phagosome acidification impairs survival inside macrophages (26) and PhoP activation (27,28). Furthermore, amino acid substitutions in PhoQ that hinder activation by mildly acidic pH attenuate virulence even though these PhoQ variants are activated normally by low Mg^2+^ and antimicrobial peptides (29). Likewise, a *ugtL* null mutant is defective for PhoQ activation by mildly acidic pH and virulence (25).

We now report that *S*. Typhimurium's ability to activate the ancestral PhoP/PhoQ system in mildly acidic pH and to cause disease requires the horizontally acquired regulatory gene *ssrB* and an SsrB binding site in the *ugtL* promoter (Figure 1). Non-pathogenic *S. bongori* fails to activate PhoQ in mildly acidic pH because it lacks both the *ssrB* gene and the SsrB binding site in the *ugtL* promoter. These findings demonstrate that horizontally acquired genes can confer new abilities by altering the properties of an ancestral regulatory system. Moreover, they suggest that foreign genes play more critical roles in evolution than previously thought. In addition, our results imply that a horizontally acquired gene may promote different phenotypes depending on the genes pre-existing in the host genome.

## MATERIALS AND METHODS

### Bacterial strains, plasmids, oligodeoxynucleotides and growth conditions

Bacterial strains and plasmids used in this study are listed in Supplemental Table S1. All *S. enterica* serovar Typhimurium strains were derived from the wild-type strain 14028s (30) and constructed by phage P22-mediated transductions (31), and the λ-red recombineering technique (32). Bacteria were grown at 37°C in Luria-Bertani (LB) broth or N-minimal media (33) supplemented with 0.1% casamino acids, 38 mM glycerol and the indicated pH (pH 7.6 or pH 4.9) and 1 mM of MgCl_2_ unless specified. *Escherichia coli* DH5α was used as the host for preparation of plasmid DNA (34). To induce plasmid expression, isopropyl β-D-1-thiogalactopyranoside (IPTG) was added at the indicated concentrations (0–1 mM). When necessary to select for plasmid maintenance, appropriate antibiotics were added at the following final concentrations: ampicillin at 50 μg ml^−1^, chloramphenicol at 20 μg ml^−1^, kanamycin at 50 μg ml^−1^ and tetracycline at 10 μg ml^−1^. DNA oligonucleotides used in this study are listed in Supplemental Table S2.

### Quantitative RT-PCR (qRT-PCR)

Total RNA was isolated using RNeasy Kit (Qiagen) and RNase-Free DNase Set (Qiagen) according to the manufacturer's instructions. The purified RNA was quantified using a Nanodrop machine (NanoDrop Technologies). Complementary DNA (cDNA) was synthesized from 1.5 μg RNA using High Capacity RNA-to cDNA Master Mix (Applied Biosystems) or SuperScript IV VILO master mix (Invitrogen), and then diluted 10-fold in water. The mRNA amounts of the *mgtC*, *ugtL*, *pcgL*, *pagC*, *pagD*, *ssaG* and *sifB* genes were determined from 2 μl of cDNA using Fast SYBR Green PCR Master Mix (Applied Biosystems) and appropriate primers (*mgtC*: 6962/6963; *ugtL*: 7295/7302; *pcgL*: 6627/6628; *pagC*: 6964/6965; *pagD*: 7016/7017; *ssrB*: 13096/13097; *ssaG*: 13098/13099; *sifB*: 17529/17530) at 0.35 μM as final concentrations, and monitored using a QuantStudio 6 machine (Applied Biosystems). Data were normalized to the levels of 16S ribosomal RNA amplified with primers 3203 and 3204 from 1000-fold diluted cDNA samples.

### Determination of *S*. Typhimurium mRNA abundance inside macrophages

The murine-derived macrophage cell line J774A.1 was cultured in Dulbecco modified Eagle medium (DMEM; Life Technologies) supplemented with 10% FBS (Life Technologies) at 37°C under 5% CO_2_. Macrophages were seeded in 6-well tissue culture plates with $2 \times {10^6}$ cells per well one day before infection with *S*. Typhimurium. Confluent monolayers were inoculated with bacterial cells that had been grown overnight in LB broth, washed with PBS and resuspended in prewarmed DMEM at a multiplicity of infection of 10. Following 30 min incubation, the wells were washed three times with prewarmed PBS to remove extracellular bacteria and then incubated with prewarmed medium supplemented with 100 µg ml^−1^ gentamicin for 1 h to kill extracellular bacteria. Next, the wells were washed three times with PBS and then incubated with prewarmed medium supplemented with 10 µg ml^−1^ gentamicin. At the indicated times, samples were harvested using TRIzol reagent (Invitrogen) solution. Total RNA was isolated and cDNA was synthesized as described above using 2 μg RNA. mRNA abundance was measured by qRT-PCR as described above. Data were normalized to the levels of 16S ribosomal RNA from twenty-fold diluted cDNA.

### Measuring gene expression by β-galactosidase assay

Bacteria with chromosomal *lacZ* fusion were grown in indicated media. β-galactosidase activity was determined as described (35). Source data can be found in Supplemental Table S3.

### Measuring gene expression by GFP assay

Bacterial cells expressing a *gfp* variant from the indicated promoter were grown in N-minimal media as described. Fluorescence and OD_600_ values were measured by using the multilabel plate reader VICTOR3 (PerkinElmer). The measured values of GFP expression were divided by 1000 times the OD_600_ values. Source data can be found in Supplemental Table S3.

### *In vivo* detection of phosphorylated PhoP

PhoP and PhoP-P were separated on 12.5% polyacrylamide gels containing acrylamide-Phos-tag™ ligand (Phos-tag™ Consortium) as described by the manufacturer. Gels were copolymerized with 50 μM Phos-tag™ acrylamide and 100 μM MnCl_2_. Whole-cell extracts were prepared as described (36) and normalized by OD_600_. The samples were electrophoresed on Phos-tag™ gels with standard running buffer [0.4% (w/v) SDS, 25 mM Tris, 192 mM glycine] at 4°C under 20 mAmp for 3.5 h, transferred to nitrocellulose membranes, and analyzed by immunoblotting using polyclonal rabbit antibodies recognizing PhoP (1:2000) and polyclonal mouse antibodies recognizing AtpB (F_0_F_1_ ATP synthase subunit A) (1:5000). Secondary horseradish peroxidase-conjugated antisera recognizing rabbit and mouse antibodies (GE healthcare) were used at 1:5000 dilution. The blots were developed with the Amersham ECL Western Blotting Detection Reagents (GE Healthcare) or SuperSignal West Femto Chemiluminescent system (Pierce), and were visualized using LAS-4000 (Fuji Film). The density of protein bands was determined by quantification using ImageJ software version 1.52 (NIH).

### Western blot analysis

Bacterial cells were grown as described and crude extracts were prepared in B-PER bacterial protein extraction reagent (Pierce) with 100 μg ml^–1^ lysozyme and EDTA-free protease inhibitor (Roche). Samples were separated in 4–12% NuPAGE gels (Life Technologies). Then, samples were analyzed by Western blotting using antibodies recognizing FLAG (Sigma; 1:2000) and AtpB (Abcam; 1:5000). Secondary horseradish peroxidase-conjugated antisera recognizing mouse antibodies (GE healthcare) were used at 1:5000 dilution. The blots were developed with the Amersham ECL Western Blotting Detection Reagents (GE Healthcare) or SuperSignal West Femto Chemiluminescent system (Pierce), and were visualized using LAS-4000 (Fuji Film). The density of protein bands was determined by quantification using ImageJ software version 1.52 (NIH).

### Chromatin immunoprecipitation

*hns-FLAG-*expressing wild-type (JC805) and *ugtL-sifB*_mu_ (JC1625) *S*. Typhimurium were grown to mid-log phase in N-minimal media with 1 mM of Mg^2+^ at pH 4.9. Bacterial cells were cross-linked with 1% formaldehyde at room temperature for 15 min, quenched with 200 mM glycine at room temperature for 10 min and washed three times with cold phosphate-buffered saline (PBS). Then, cells were lysed in cell lysis solution A [10 mM Tris, pH 8.0, 50 mM NaCl, 10 mM EDTA, 20% sucrose, 10 mg ml^−1^ lysozyme] and 10× RIPA solution (Millipore). DNA was fragmented to average size of 500 bp by sonication (VirTis) and a 50 μl aliquot was taken as input DNA. Immunoprecipitation of complexes between the heat stable nucleoid-structuring (H-NS) protein and DNA were performed using antibodies recognizing FLAG, and using MagnaChip protein A/G magnetic beads (Millipore). Samples were then washed twice with 1× RIPA solution, twice with LiCl immune complex wash buffer (Millipore), twice with TE buffer [20 mM Tris (pH 8.0), 1 mM EDTA], and eluted in elution buffer [50 mM Tris (pH 8.0), 10 mM EDTA, 1% SDS] with incubation at 65°C for 15 min. Both immunoprecipitated (IP) and input DNA samples were incubated at 65°C for 9 h to reverse crosslinks, purified using Qiagen PCR purification column, and were quantified using qRT-PCR using primers (*mgtA*: 7225/7226; *ugtL*: 7295/7302; *sifB*: 17529/17530; *STM14_3310*: 16879/16880; *rpoD*: 4149/4150). Binding of H-NS protein to DNA were calculated as ratio of ‘IP DNA/input DNA’ and normalized to that of *rpoD*.

### Purification of the SsrBc protein

To purify the SsrBc protein, *E. coli* BL21 (DE3) harboring a plasmid expressing His6-tagged SsrB C-terminus portion (pH6-SsrBc) was grown in LB at 37°C for 3 h and 0.7 mM of IPTG was added to induce gene expression and further incubated at 30°C for 3 h. Cells were collected and washed twice with a solution containing 50 mM Tris–HCl (pH 8.0) and 150 mM NaCl. Washed cells were resuspended in solution A [50 mM Tris–HCl (pH 8.0), 150 mM NaCl] containing 150 μg ml^−1^ lysozyme, 1 mM MgCl_2_, DNase I (Promega) and protease EDTA-free protease inhibitor (Roche), and incubated at 4°C for 30 min. Cells were broken using Cell Disruptor (Constant Systems Ltd). After adding imidazole to 20 mM as final concentration, cell debris was removed by centrifugation (12 000 × *g*, 30 min) and the supernatant was applied to Ni-Nta agarose (Qiagen) column. The column was washed with solution A containing 25 mM imidazole, and proteins were eluted with solution A containing 100–300 mM imidazole and dialyzed with the same solution without imidazole. The purified SsrBc protein was separated in 4–12% NuPAGE gels (Life Technologies) and visualized by coomassie G-250 staining (Thermo Scientific) (Supplemental Figure S1), demonstrating purity of ∼94%.

### Electrophoretic mobility shift assay

DNA fragments corresponding the *ugtL-sifB* intergenic region and the 3′ end of *purB* coding region with the *purB-phoP* intergenic region were generated by PCR from wild-type *S*. Typhimurium (14028s) using primers 17222/17223 and 14244/14217, respectively. The DNA fragments were gel purified with QIAquick column (Qiagen). Purified probe DNA (2 or 4 nM) and purified SsrBc protein at indicated concentrations (0–2 μM) were mixed with binding buffer [20 mM HEPES (pH 8.0), 10 mM KCl, 2 mM MgCl_2_, 0.1 mM EDTA, 0.1 mM DTT, 50 μg ml^−1^ BSA, 10% (vol/vol) glycerol, and 10 ng μl^−1^ of poly(dI-dC) (Sigma)] in a total volume of 20 μl and incubated for 15 min at room temperature. Samples were then electrophoresed on 6% Tris-borate-EDTA gels (Life Technologies) and stained using EMSA kit (Invitrogen) according to the manufacturer's instructions.

### DNase I footprint assay

DNA fragments corresponding to the 3′ end of the *purB* coding region with the *purB-phoP* intergenic region were generated by PCR from wild-type *S*. Typhimurium (14028s) using primers 14244 and ^32^P-labeled 14217. The DNA fragments were gel purified with QIAquick column (Qiagen). A total of ∼10^5^ cpm of labeled DNA probe (∼2 nM) and purified SsrBc (0, 0.25 and 1 μM) were mixed with the same binding buffer used in the electrophoretic mobility shift assay including 50 ng μl^−1^ of poly(dI-dC) (Sigma) in a total volume of 20 μl and incubated for 15 min at room temperature. DNase I (Promega) (0.01 units), 10 mM CaCl_2_, and 10 mM MgCl_2_ were added and incubated for 3 min at room temperature. The reaction was stopped by the addition of 100 μl of phenol chloroform, and the aqueous phase was precipitated with ethanol. The precipitate was dissolved in sequence-loading buffer and electrophoresed on a 6% acrylamide/7 M urea gel together with a sequence ladder initiated with the labeled primer by using the T7 Sequenase 2.0 DNA-sequencing kit (Amersham Biosciences), and the gels were dried and autoradiographed (Fujifilm).

### Mouse virulence assay

Six-week-old female BALB/c or C3H/HeN mice were purchased from Charles River Laboratories. Five mice in each group were infected intraperitoneally with 0.1 ml of PBS containing ∼10^2^ (for BALB/c) or ∼2 × 10^4^ (for C3H/HeN) *S*. Typhimurium that had been grown overnight in LB broth and resuspended and diluted in PBS. Mouse survival was monitored every 12 h for 3 weeks. Virulence assays were conducted twice with similar outcomes, and data for each experimental group correspond to groups of five mice. All animals were housed in temperature- and humidity-controlled rooms and maintained on a 12 h light/12 h dark cycle. All procedures complied with regulations of the Institutional Animal Care and Use Committee of the Yale School of Medicine.

### Construction of chromosomal mutant strains

To generate a mutant strain expressing the *ugtL* gene from the heterologous promoter p*_lac_*_1–6_, a *cat* cassette with p*_lac_*_1–6_ was introduced upstream of the *ugtL* leader region to replace the *ugtL* promoter. That is, the *cat* fragment with the p*_lac_*_1–6_ was amplified from plasmid pKD3 using primers 16655/16658, then introduced into wild-type *S*. Typhimurium (14028s; *S*. Typhimurium) harboring plasmid pKD46 (32). The *cat* cassette was removed using plasmid pCP20 (32).

To generate strains deleted for SPI-2 genes, a *kan* cassette was introduced in the indicated region of SPI-2: the *kan* fragment was amplified from plasmid pKD13 using primers 13160/13161 (for deleting *sseA–sseG* genes), 13160/13165 (for deleting *sseA-ssaU* genes), 13164/13165 (for deleting *ssaG-ssaU* genes) then introduced into wild-type *S*. Typhimurium (14028s) harboring plasmid pKD46 (32).

To generate a strain harboring *ugtL-FLAG*, a *kan* cassette was introduced in the 3’end of the *ugtL* gene as follows: the *kan* fragment with the FLAG coding sequence was amplified from plasmid pKD4 using primers 16686/16687, then introduced into wild-type *S*. Typhimurium (14028s) harboring plasmid pKD46 (32).

To generate a strain with mutated SsrB binding region in the *ugtL-sifB* intergenic region, a cassette with *cat* and P*rhaB-relE* was introduced into the region containing a putative SsrB binding site: the *cat* P*rhaB-relE* fragment was amplified from plasmid pSLC-242 (37) using primers 17220/17221, then introduced into wild-type *S*. Typhimurium (14028s) harboring plasmid pKD46 (32). Then, the *cat* P*rhaB-relE* cassette was replaced by annealed oligonucleotides 17234/17235 to remove SsrB binding site. The resulting strain was obtained following selection against RelE-mediated toxicity on media containing 0.2% rhamnose as described (37). Mutation was confirmed by DNA sequencing.

To generate *S. bongori* expressing the *phoQ* gene from *S*. Typhimurium (*phoQ*^ST^), a *tetRA* cassette was amplified using primers 14810/14811 from transposon Tn*10* in strain MS7953s and was introduced into the *phoQ* gene of wild-type *S. bongori* (S3041) harboring pKD46 (32). The *phoQ* gene of *S*. Typhimurium was amplified using primers 14821/14822 and genomic DNA of strain 14028s. The *tetAR* cassette was replaced by a PCR product to create *S. bongori* with *phoQ*^ST^. The resulting strain was selected against tetracycline resistance on media containing fusaric acid (38), and the gene replacement was confirmed by DNA sequencing.

To generate a strain with mutated SsrB binding site in the region upstream of the *phoP* gene (located in the *purB* gene), the SsrB binding sequence was mutated and a copy of intact *purB* gene and upstream gene promoter (P*ycfC*) were integrated into the attachment Tn*7* site. The normal *purB* gene was deleted except the portion containing the SsrB binding site. The engineering of these chromosomal mutations was carried out as follows: a *tetRA* cassette was amplified by PCR using primers 14950/14951 from transposon Tn*10* in strain MS7953s, then introduced into wild-type *S*. Typhimurium (14028s) harboring plasmid pKD46 (32). The resulting strain was grown in media containing 0.5 mM adenine. The *tetRA* cassette was then replaced with annealed oligonucleotides 14952/14953 to mutate SsrB binding site. The resulting strain was obtained following selection against tetracycline resistance on media containing fusaric acid (38), and the mutation was confirmed by DNA sequencing. Then, the P*ycfC* and *purB* gene were introduced into the attachment Tn*7* site: the P*ycfC* and *purB* gene were amplified by PCR from wild-type *S*. Typhimurium (14028s) genomic DNA using primers 16540/16541, 16542/16543, then introduced into pGRG36 (digested with XmaI and XhoI) by Gibson assembly. The resulting plasmid was verified by DNA sequencing and introduced into the attachment Tn*7* site as described (39). Integration of the P*ycfC*-*purB* gene and its upstream gene promoter was verified by DNA sequencing. Then, a *kan* cassette was introduced into the *purB* gene at normal chromosomal location: the *kan* cassette was amplified from plasmid pKD4 using primers 16869/16870, then introduced into *S*. Typhimurium strains with the P*ycfC*-*purB* fragment at the attachment Tn*7* site and wild-type or mutated SsrB binding site in the upstream region of the *phoP* gene harboring plasmid pKD46 (32). The *kan* cassette was removed using plasmid pCP20 (32).

### Construction of plasmids

Plasmid pSsrB was constructed as follows: the *ssrB* gene was amplified from wild-type *S*. Typhimurium (14028s) using primers 14225/14226, then introduced between the BamHI and HindIII sites of pUHE21–2*lacI*^q^ (40).

Plasmid pSsrB^V197A^ was constructed by site-directed mutagenesis using the Quikchange lightning site-directed mutagenesis kit with primers 15755/15756 and plasmid pSsrB as a template following manufacturer's instructions.

Plasmid pH6-SsrBc was constructed as follows: the *ssrB* gene was amplified from wild-type *S*. Typhimurium (14028s) using primers 14224/14225, then introduced between the BamHI and HindIII sites of pUHE21–2*lacI^q^* (40).

Plasmid pUgtL^SB^ was constructed as follows: the *ugtL* gene was amplified from wild-type *S. bongori* (S3041) using primers 16058/16059, then introduced between the BamHI and HindIII sites of pUHE21–2*lacI^q^*(40).

Plasmid pFPV25::PpmrD-GFPaav, pFPV25::Pmig-14-GFPaav were constructed as follows: the *pmrD* and *mig-14* promoters were amplified from wild-type *S*. Typhimurium (14028s) using primers 5900/4740 and 4803/6044, then introduced between the EcoRI and BamHI sites of pFPV25AAV (41).

### Protein and nucleotide sequence comparisons

Amino acid sequence and the upstream region of DNA in the indicated strains were compared to those of *S*. Typhimurium 14028s using TBLASTN/BLASTN and Clustal Omega (EMBL-EBI). A phylogenetic tree was generated by interactive Tree of Life software (v5) based on the analysis of the *ugtL* gene and its upstream region containing the SsrB binding site (720 nt upstream from the start codon (AUG) of the *ugtL* gene and whole coding region).

### Statistical analyses

Sample sizes (biological replicates) for each experimental group or condition are described in each figure legend. For comparisons of two groups, *t*-tests were applied. Two-sided analysis provides *P*-values for each comparison. For comparisons of more than two groups, one-way ANOVA with Brown-Forsythe and Welch tests were applied. Each group was compared with a control group (wild-type unless specified).

## RESULTS

### Non-pathogenic *S. bongori* harbors a functional *ugtL* virulence gene

The PhoP/PhoQ systems of *S. bongori* and *S*. Typhimurium differ in that the former is activated by low Mg^2+^ and antimicrobial peptides, but not mildly acidic pH (29), whereas the latter is activated by all three signals (29,42–44). This disparity is due, in part, to differences between their PhoQ proteins because a *S*. Typhimurium strain expressing the *S. bongori phoQ* gene instead of its own was defective for activation by mildly acidic pH but not by low Mg^2+^ and antimicrobial peptides (29).

Unexpectedly, we found that a *S. bongori* strain expressing the *S*. Typhimurium *phoQ* gene instead of its own was also defective for activation by mildly acidic pH but not by low Mg^2+^ (Supplemental Figure S2). This result indicates that an additional factor(s) is responsible for the disparate abilities of the two *Salmonella* species to activate the PhoP/PhoQ system in mildly acidic pH.

**Figure 2.**
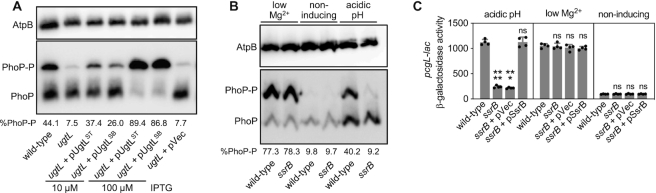
SsrB is necessary for PhoP activation, which requires UgtL, in mildly acidic pH. (A, B) Phos-tag western blot analysis of crude extracts prepared from (**A**) wild-type (JC805) and *ugtL* (JC925) *S*. Typhimurium strains with indicated plasmids (pVec, empty vector; pUgtL^ST^, plasmid expressing *S*. Typhimurium *ugtL*; pUgtL^SB^, plasmid expressing *S. bongori ugtL*) grown to mid-log phase in N-minimal media with 1 mM of Mg^2+^ at pH 4.9 supplemented with indicated concentrations of IPTG, and (**B**) wild-type (14028s) and *ssrB* (EG14411) *S*. Typhimurium strains grown to mid-log phase in N-minimal media with 1 mM of Mg^2+^ at pH 4.9 (acidic pH), 10 μM Mg^2+^ at pH 7.6 (low Mg^2+^), or 1 mM Mg^2+^ at pH 7.6 (non-inducing) using antibodies recognizing PhoP or the loading control AtpB. Representatives of at least three independent experiments are shown. Numbers under the blots indicate % phosphorylated PhoP (PhoP-P). (**C**) β-galactosidase activity produced from a chromosomal *pcgL-lacZ* fusion in wild-type (EG9331) and *ssrB* (JC201) *S*. Typhimurium with an empty vector (pVec) or a plasmid expressing SsrB (pSsrB) grown to mid-log phase in N-minimal media with 1 mM of Mg^2+^ at pH 4.9 (acidic pH), 10 μM Mg^2+^ at pH 7.6 (low Mg^2+^), or 1mM Mg^2+^ at pH 7.6 (non-inducing) supplemented with 0.2 mM IPTG. The mean and SD from four independent experiments are shown (n = 4). Each dot represents individual biological sample. One-way ANOVA with Brown-Forsythe and Welch tests (wild-type vs. others); ns, not significant; ***P < 0.001, ****P < 0.0001.

We reasoned that the *ugtL* gene might be that factor given that the deduced amino acid sequences of the *ugtL* genes share only 55% identity (Supplemental Figure S3), which is much lower than most proteins present in both species (e.g. the PhoQ proteins are 99% identical). However, a plasmid expressing the *S. bongori ugtL* gene from a heterologous promoter increased the fraction of PhoP-P in a *S*. Typhimurium *ugtL* mutant as much as the isogenic plasmid with the *S*. Typhimurium *ugtL* gene (Figure 2A). A 10-fold increase in the inducer concentration used to activate the heterologous promoter driving *ugtL* transcription resulted in the quasi-complete conversion of PhoP into PhoP-P (Figure 2A). By contrast, the plasmid vector had no effect (Figure 2A). These results demonstrate that the *S. bongori ugtL* gene is competent for activation of the *S*. Typhimurium PhoP/PhoQ system in mildly acidic pH. Why, then, does mildly acidic pH activate PhoP/PhoQ in *S*. Typhimurium but not in *S. bongori* even though both species have functional *ugtL* genes?

### PhoP activation in mildly acidic pH requires the regulatory gene *ssrB*

*S. bongori* lacks the horizontally acquired SPI-2 (12) and is unable to activate PhoP/PhoQ in mildly acidic pH (Supplemental Figure S2) (29). Because the SPI-2-encoded SsrB protein is activated in mildly acidic pH (16,45), like PhoP (27,29,44), we investigated the possibility of the *ssrB* gene being required for activation of the PhoP/PhoQ system when *S*. Typhimurium experiences mildly acidic pH.

We determined that the fraction of PhoP-P was much lower in the *ssrB* mutant than in wild-type *S*. Typhimurium when bacteria were grown in mildly acidic pH (pH 4.9, 1 mM Mg^2+^) (Figure 2B), mimicking the behavior of the *ugtL* null mutant (Figure 2A) (25). By contrast, the fraction of PhoP-P was almost the same in isogenic wild-type and *ssrB S*. Typhimurium when grown in low Mg^2+^ (pH 7.6, 10 μM Mg^2+^) (Figure 2B), an inducing condition that activates PhoP as much as mildly acidic pH in a *ugtL*-independent manner (25,29). Under non-inducing conditions, PhoP-P was not detected in wild-type or *ssrB S*. Typhimurium (Figure 2B). These results demonstrate that *ssrB* is necessary to activate PhoP when the PhoQ-activating condition is mildly acidic pH.

The *ssrB*-dependent activation of PhoP taking place in mildly acidic pH is necessary for PhoP-dependent gene transcription because the β-galactosidase activity from the PhoP-activated *pcgL-lac* transcriptional fusion was much lower in the *ssrB* mutant than in the isogenic *ssrB^+^* strain (Figure 2C), reflecting the abundance of PhoP-P (Figure 2B) (46). By contrast, the two strains had similarly high β-galactosidase activity when grown in low Mg^2+^ (Figure 2C) and similarly low β-galactosidase activity when grown in non-inducing conditions (Figure 2C). A plasmid with a wild-type copy of the *ssrB* gene complemented the *ssrB* mutant, whereas the plasmid vector did not (Figure 2C). The *ssrB* gene is required for normal transcription of other PhoP-activated genes because the *ssrB* mutant exhibited lower expression of PhoP-activated genes in strains harboring a *lac* transcriptional fusion to the *ugtL* gene (Supplemental Figure S4A), *gfp* transcriptional fusions to the *phoP, pmrD*, and *mig-14* genes (Supplemental Figure S4B), and also when examining the mRNA amounts of the *mgtC, ugtL, pcgL, pagC* and *pagD* genes (Supplemental Figure S4C), than the isogenic *ssrB^+^* strain (40,42,46). Thus, the defect of the *ssrB* mutant is neither specific to a particular PhoP-activated gene nor to the reporter used.

SsrB appears to activate PhoP by regulating expression of a gene(s) outside of SPI-2 because the fluorescence from a *phoP-gfp* fusion was not altered upon deletion of 26 of 30 genes in SPI-2 (Supplemental Figure S4D). Taken together, the results in this section indicate that when *S*. Typhimurium experiences mildly acidic pH, the horizontally acquired *ssrB* gene activates the ancestral regulator PhoP, resulting in transcription of PhoP-activated genes.

### SsrB activates PhoP in a *phoQ*-dependent manner

**Figure 3.**
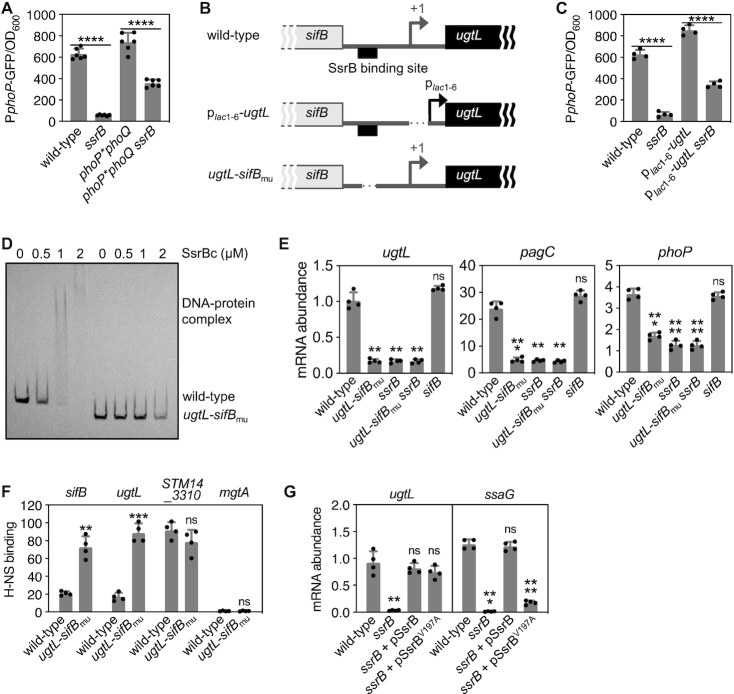
SsrB promotes UgtL expression, thereby enhancing PhoP activation in mildly acidic pH. (**A, C**) Fluorescence produced from a P*phoP-gfp* transcriptional fusion displayed by (**A**) wild-type (14028s), *ssrB* (EG14411), phoP*phoQ (EG10232), and phoP*phoQ ssrB (JC449) or (**C**) wild-type (14028s), *ssrB* (EG14411), p*_lac_*_1–6_*-ugtL* (JC1360), p*_lac_*_1–6_*-ugtL ssrB* (JC1449) *S*. Typhimurium. Bacteria were grown to mid-log phase in N-minimal media with 1 mM of Mg^2+^ at pH 4.9. (**B**) Schematics of the intergenic region between the *ugtL* and *sifB* genes in wild-type, p*_lac_*_1–6_-*ugtL* and *ugtL-sifB*_mu_*S*. Typhimurium strains. (**D**) *In vitro* binding of SsrBc to the wild-type or mutant (mu) *ugtL-sifB* intergenic region DNA. Four nM of the *ugtL-sifB* intergenic region DNA (wild-type or mu) was incubated with purified SsrBc (0, 0.5, 1 and 2 μM) proteins. A representative of at least three independent experiments is shown. (**E**) mRNA abundance of the *ugtL, pagC* and *phoP* genes produced by wild-type (14028s), *ugtL-sifB*_mu_ (JC1547), *sifB* (JC1567), *ugtL-sifB*_mu_*ssrB* (JC1548), and *ssrB* (EG14411) *S*. Typhimurium grown to mid-log phase in N-minimal media with 1 mM of Mg^2+^ at pH 4.9. (**F**) *In vivo* binding of H-NS to the promoter regions of the *sifB*, *ugtL*, *STM14_3310* and *mgtA* genes were determined in *hns-FLAG* (JC805) and *hns-FLAG ugtL-sifB*_mu_ (JC1625) *S*. Typhimurium strains grown to mid-log phase in N-minimal media with 1 mM of Mg^2+^ at pH 4.9 using chromatin immunoprecipitation. (**G**) mRNA abundance of the *ugtL* and *ssaG* genes produced by wild-type (14028s) and *ssrB* (EG14411) *S*. Typhimurium with plasmids expressing wild-type SsrB or its variant (V197A) grown to mid-log phase in N-minimal media with 1 mM of Mg^2+^ at pH 4.9 (acidic pH) supplemented with 5 μM IPTG. The mean and SD from at least four independent experiments are shown (A, *n* = 6; C, E–G, *n* = 4). Each dot represents individual biological sample (A, C, E–G). Two-tailed *t*-test with (A and C) *ssrB^+^* versus*ssrB^−^* or (F) wild-type versus *ugtL-sifB*_mu_. One-way ANOVA with Brown-Forsythe and Welch tests (wild-type versus others) (E and G); ns, not significant; **P < 0.01, ***P < 0.001, ****P < 0.0001.

We hypothesized that PhoP activation by SsrB requires PhoQ because this sensor is the only known PhoP phosphodonor (47) and SsrB promotes the phosphorylated state of PhoP (Figure 2B). In agreement with this notion, fluorescence from *phoP-gfp* was largely similar (i.e. 2-fold difference) in a strain with a *phoQ* null allele and specifying a PhoP variant capable of autophosphorylation from acetyl phosphate (47) and the isogenic *ssrB* null mutant (Figure 3A). By contrast, fluorescence was 10 times higher in wild-type *S*. Typhimurium than in the *ssrB* mutant (Figure 3A and Supplemental Figure S4B). Thus, SsrB activation of PhoP is largely PhoQ-dependent. (The two-fold *phoQ-*independent effect is addressed below under **SsrB promotes *phoP* transcription by binding to the *purB* coding region upstream of the *phoP* promoter**.)

### SsrB activates PhoP by promoting transcription of the *ugtL* gene

We posit that SsrB activates PhoP in mildly acidic pH by promoting *ugtL* transcription because *ugtL* is necessary for PhoQ-dependent phosphorylation of PhoP in mildly acidic pH (25), and also because SsrB promotes transcription of horizontally acquired genes outside of SPI-2 (17–19) and *ugtL* is a horizontally acquired gene (48). As proposed, the amounts of the *ugtL* mRNA and UgtL protein were higher in wild-type *S*. Typhimurium than in the *ssrB* mutant (Supplemental Figures S4C and S5). Moreover, when *ugtL* transcription was driven from the constitutive p*_lac_*_1–6_ promoter (49) (Figure 3B), isogenic *ssrB* strains displayed similar fluorescence from *phoP-gfp* (Figure 3C) (i.e., ∼2-fold difference. The two-fold *ugtL* promoter*-*independent effect is addressed below under **SsrB promotes *phoP* transcription by binding to the *purB* coding region upstream of the *phoP* promoter**). In other words, *ugtL* transcription from a constitutive promoter largely bypassed the SsrB requirement for PhoP activation. How, then, does SsrB promote *ugtL* transcription in wild-type *S*. Typhimurium?

A previous study showed SsrB binding to the intergenic region that separates the divergently transcribed *ugtL* and *sifB* genes (Figure 3B) (17). This region is absent from the equivalent region of the *S. bongori* genome (Supplemental Figure S6), which has neither *sifB* (Supplemental Figure S6) nor *ssrB* (12). The identified region has a *bona fide* SsrB binding site because the purified DNA binding domain of SsrB (19) bound to a DNA fragment harboring this region but not to one deleted for it (Figure 3D). Moreover, the site is necessary for PhoP activation in mildly acidic pH because the mRNA abundances of the PhoP-activated *pagC* and *phoP* genes were lower in an engineered strain deleted for the SsrB binding site (Figure 3B) than in the isogenic wild-type strain (Figure 3E). Independent support for this notion was obtained when examining bacteria carrying the *phoP-gfp* fusion: fluorescence was ∼5-fold lower in the SsrB binding site mutant than in the isogenic wild-type strain (Supplemental Figure S7A).

The defective PhoP activation exhibited by the SsrB binding site mutant is due to the inability of the SsrB protein to promote *ugtL* expression. This is because the SsrB binding site mutant produced the same low *ugtL* mRNA amounts as a mutant lacking the *ssrB* gene and a double mutant lacking both *ssrB* and the SsrB binding site (Figure 3E). By contrast, a strain deleted for the *sifB* gene retained wild-type *ugtL* mRNA amounts (Figure 3E), indicating that the phenotype of the SsrB binding site mutant is not due to compromised *sifB* expression.

When low Mg^2+^ was the activating signal for PhoQ, the SsrB binding site mutant retained wild-type expression of the *phoP* and *ugtL* genes (Supplemental Figure S7). This result is congruent with *ssrB* being dispensable for PhoP activation during growth in low Mg^2+^ (Figure 2B). Critically, *ugtL* transcription from a heterologous promoter bypasses the SsrB requirement (Supplemental Figure S8) but not the need for a mildly acidic pH (Supplemental Figure S8) for activation of the PhoP protein. Therefore, PhoP activation in mildly acidic pH requires PhoQ’s intrinsic ability to respond to a mildly acidic pH. In sum, the SsrB binding site in the *ugtL* promoter region is specifically required for PhoP activation in mildly acidic pH.

### SsrB antagonizes the gene silencer H-NS in the *ugtL* promoter region

The xenogeneic silencer H-NS binds to AT-rich horizontally acquired DNA, preventing expression of the corresponding genes (50,51), including *ugtL* and *sifB*. Given that SsrB overcomes the silencing effects of H-NS at certain horizontally acquired genes (52), we wondered whether SsrB promotes *ugtL* transcription by binding to the SsrB binding site in the *ugtL-sifB* intergenic region that is bound by H-NS. Using chromatin immunoprecipitation, we determined that H-NS bound to the *ugtL-sifB* region more strongly in the strain with the mutant SsrB binding site than in the isogenic wild-type strain (Figure 3F). Control experiments showed that the SsrB binding site mutant retained normal H-NS binding to the *STM14_3310* DNA (Figure 3F), one of the genes most tightly bound by H-NS (50), and no detectable binding to the DNA region upstream of *mgtA* (Figure 3F), a PhoP-activated gene not bound by H-NS (50).

SsrB appears to promote *ugtL* transcription solely by antagonizing silencing by H-NS (as opposed to being required to recruit RNA polymerase) because *ugtL* mRNA abundance was similar in isogenic *ssrB* mutant strains harboring plasmids expressing either the wild-type SsrB protein or the SsrB (V197A) variant (Figure 3G), which retains wild-type DNA binding ability but is impaired in RNA polymerase recruitment (53). Control experiments showed defective *ssaG* transcription in the strain expressing the SsrB (V197A) variant (Figure 3G), in agreement with previous results demonstrating that *ssaG* transcription by SsrB requires RNA polymerase recruitment (53). (Please note that it is not possible to genetically test the participation of *hns* in *ugtL* expression because *hns* is an essential gene in *S*. Typhimurium (50), and also because suppressors that allow growth of an *hns* mutant fail to grow under the virulence-relevant conditions necessary to activate the SsrB protein.) Taken together, the results presented in this section suggest that SsrB promotes *ugtL* transcription by displacing H-NS from the *ugtL-sifB* intergenic region.

### SsrB promotes *phoP* transcription by binding to the *purB* coding region upstream of the *phoP* promoter

SsrB appears to activate PhoP by means other than promoting *ugtL* transcription because inactivation of the *ssrB* gene decreases expression of the PhoP-activated *phoP* promoter in a strain in which the *ugtL* gene is transcribed from a heterologous promoter (Figure 3C), and also because the *ssrB* mutant exhibited defective *phoP* expression in a phoP* phoQ strain (Figure 3A) even though UgtL activates PhoP in a PhoQ-dependent manner (25). As detailed below, we have now established that SsrB is a direct transcriptional activator of the *phoP* gene.

**Figure 4.**
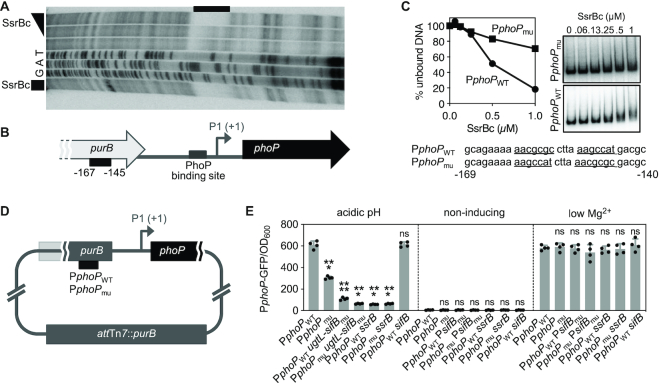
SsrB promotes *phoP* transcription by binding to the *purB* coding region upstream of the *phoP* promoter. (**A**) DNase I footprinting analysis of the *phoP* promoter using purified SsrBc-His6 protein (0, 0.25 and 1 μM, triangle; 1 μM, square). Lanes G, A and T correspond to dideoxy chain-termination sequences for the *phoP* promoter DNA. The black bar indicates the region protected by the SsrBc-His6 protein. (**B**) Schematic of the *purB-phoP* chromosomal region. The SsrB binding site is located in the coding region of the *purB* gene. Numbers under the black box indicate distance from the +1 (P1, PhoP-activated transcriptional start site). (**C**) *In vitro* binding of SsrBc-His6 to the wild-type or mutant SsrB binding site DNAs. DNA (2 nM) was incubated with SsrBc-His6 proteins (0, 0.06, 0.13, 0.25, 0.5 and 1 μM). A representative of at least three independent experiments is shown. Numbers underneath the nucleotides indicate distance from P1. (**D**) Schematic of strains with wild-type (P*phoP*_WT_) or mutated (P*phoP*_mu_) SsrB binding site in the *purB* coding region (on the *purB* gene which is inactivated) and a copy of *purB* gene integrated into attachment Tn*7* site. (**E**) Fluorescence produced from a P*phoP-gfp* transcriptional fusion displayed by P*phoP*_WT_ (JC1482), P*phoP*_mu_ (JC1458), P*phoP*_WT_*ugtL-sifB*_mu_ (JC1582), P*phoP*_mu_*ugtL-sifB*_mu_ (JC1583), P*phoP*_WT_*ssrB* (JC1463), P*phoP*_mu_*ssrB* (JC1464), and P*phoP*_WT_*sifB* (JC1586) *Salmonella* grown to mid-log phase in N-minimal media with 1 mM of Mg^2+^ at pH 4.9 (acidic pH), 10 μM Mg^2+^ at pH 7.6 (low Mg^2+^), or 1 mM Mg^2+^ at pH 7.6 (non-inducing). The mean and SD from four independent experiments are shown. One-way ANOVA with Brown-Forsythe and Welch tests (P*phoP*_WT_ vs. others); ns, not significant; ***P < 0.001, ****P < 0.0001.

DNase I footprinting analysis of the *phoP* promoter region (∼300 bp) demonstrated that the purified DNA binding domain of SsrB protects a region 145–167 nt upstream of the PhoP-dependent transcription start site (P1) of the *phoP* gene (Figure 4A and 4B). This region is located within the *purB* coding region (Figure 4B) and resembles other SsrB binding sites (17). Nucleotide substitutions in the identified SsrB binding site (Figure 4C and 4D) hampered SsrB binding *in vitro* (Figure 4C), and reduced *phoP* transcription *in vivo* in bacteria experiencing a mildly acidic pH (Figure 4E). (Because the nucleotide substitutions in the SsrB binding site disrupt the *purB* coding region, these experiments were carried out using isogenic strains harboring a wild-type copy of the *purB* gene integrated into the attachment Tn*7* site (Figure 4D).) A double mutant with nucleotide substitutions in the SsrB binding site in the *purB* coding region upstream of the *phoP* promoter and the deletion of the SsrB binding region in the *ugtL* promoter was as defective in PhoP-dependent transcription as the *ssrB* null mutant (Figure 4E). The effect of the nucleotide substitutions in the SsrB binding sites and *ssrB* gene are specific to mildly acidic pH because the corresponding mutants behaved like the isogenic wild-type strain when experiencing low Mg^2+^ or non-inducing conditions (Figure 4E). Cumulatively, these results indicate that SsrB activates PhoP by promoting transcription of both the *phoP* and *ugtL* genes.

### SsrB-dependent activation of the *ugtL* gene is necessary for virulence

*S*. Typhimurium's ability to cause disease in a mouse model of infection requires functional *phoP*, *phoQ* (21,22), *ugtL* (25) and *ssrB* (54) genes, as well as PhoQ’s ability to respond to mildly acidic pH (27,29,55). To determine whether the SsrB-dependent activation of PhoP in mildly acidic pH is necessary for virulence, we compared a set of isogenic strains with a wild-type or defective SsrB binding site in the *ugtL-sifB* intergenic region (*ugtL-sifB*_mu_; Figure 3B), and, as controls, mutants lacking the *ssrB* or *sifB* genes.

**Figure 5.**
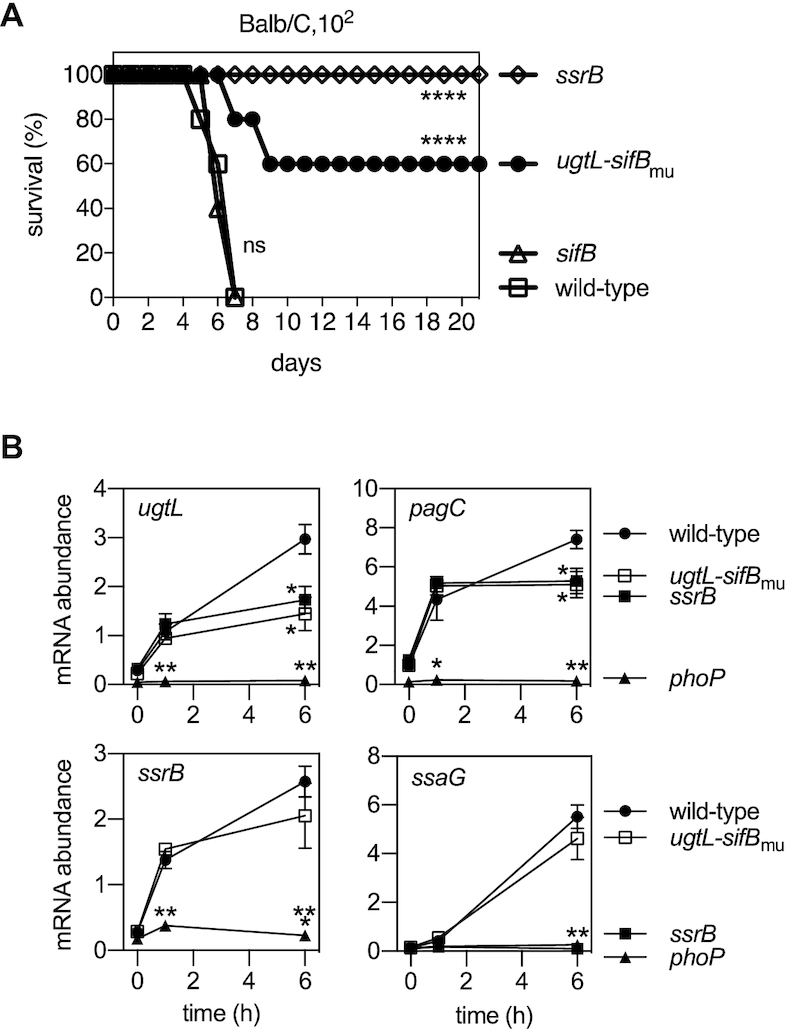
SsrB-dependent activation of *ugtL* transcription is required for *S*. Typhimurium virulence in mice and for transcription of PhoP-activated genes inside macrophages. (**A**) Survival of BALB/c mice inoculated intraperitoneally with ∼10^2^ wild-type (14028s), *ugtL-sifB*_mu_ (JC1547), *sifB* (JC1567) and *ssrB* (EG14411) *S*. Typhimurium. Data are representative of two independent experiments, which produced similar results, *n* = 5 mice per each experimental group. Mantel-Cox test was performed between wild-type and isogenic mutant *Salmonella* infected mice; ns, not significant, ****P < 0.0001. (**B**) mRNA abundance of the *ugtL*, *pagC*, *ssrB* and *ssaG* genes produced by wild-type (14028s), *phoP* (MS7953s), *ssrB* (EG14411) and *ugtL-sifB*_mu_ (JC1547) *Salmonella* harvested from the macrophage-like cell line J774A.1 at the indicated times. The mean and SD from three independent experiments are shown. One-way ANOVA with Brown-Forsythe and Welch tests (wild-type vs. others) were applied at each time point; no *, not significant, *P < 0.05, **P < 0.01, ***P < 0.001.

When inoculated via the intraperitoneal route, the SsrB binding site mutant was attenuated in both C3H/HeN and Balb/C mice (Figure 5A and Supplemental Figure S9). (A salient difference between these mouse strains is that the former has two functional copies of the *Slc11a1* gene, whereas the latter has two defective copies of it. Although *Slc11a1* confers resistance to *S*. Typhimurium (56), *phoP* and *phoQ* single mutants are attenuated for virulence in both C3H/HeN and Balb/C mice (21–23,25,29).) The virulence attenuation of the SsrB binding site mutant (*ugtL-sifB*_mu_) is due to impaired expression of *ugtL* (as opposed to *sifB*) because the *sifB* null mutant displayed wild-type virulence (Figure 5A and Supplemental Figure S9), as reported (57). The SsrB binding site mutant (*ugtL-sifB*_mu_) was not as attenuated as the *ssrB* null mutant (Figure 5A and Supplemental Figure S9), in agreement with SsrB being required for expression of virulence genes in addition to *ugtL* (16,17,19).

### PhoP and SsrB exhibit different activation kinetics when *S*. Typhimurium is inside macrophages

We hypothesized that the virulence defect of the SsrB binding site mutant (*ugtL-sifB*_mu_) (Figure 5A and Supplemental Figure S9) is due to diminished PhoP activation inside macrophages because PhoP is activated when *S*. Typhimurium is in a mildly acidic macrophage phagosome (27,28) and also because PhoP activation in mildly acidic pH is necessary for *S*. Typhimurium virulence (25,27,29). To test this hypothesis, we examined the mRNA abundance of PhoP-activated genes at different times after *S*. Typhimurium internalization by a murine macrophage-like cell line.

The mRNA abundance of the PhoP-activated *ugtL, pagC* and *ssrB* genes was similar among wild-type, *ugtL-sifB*_mu_ mutant and *ssrB* null strains at 1 h post bacterial internalization by macrophages (Figure 5B). By contrast, the mRNA abundance of *ugtL* and *pagC* genes was lower in both the *ugtL-sifB*_mu_ mutant and the *ssrB* null strain than in wild-type *S*. Typhimurium at 6 h post bacterial internalization by macrophages (Figure 5B). (*ssrB* mRNA amounts were similar in the *ugtL-sifB*_mu_ mutant and wild-type strain (Figure 5B).) The *phoP* null mutant had low mRNA amounts for all three genes at both times (Figure 5B). Therefore, PhoP activation is *ssrB-*dependent at 6 h but -independent at 1 h inside macrophages.

The behavior of the *ugtL* and *pagC* genes is in contrast to that of *ssrB* and the SsrB-activated *ssaG* gene (58). That is, there was little *ssaG* expression at 1 h (Figure 5B), and the mRNA at 6 h accumulated in a *ssrB-* and *phoP-*dependent manner (Figure 5B). The *ugtL-sifB*_mu_ mutant was minimally defective at 6 h post bacterial internalization (Figure 5B). Although the *phoP* mutant is defective in *ssaG* expression at 6 h inside macrophages (Figure 5B), it behaves like the wild-type strain in bacteria grown in defined laboratory media of mildly acidic pH (Supplemental Figure S10). These results indicate that PhoP activation occurs early on, and SsrB activation takes place later when *S*. Typhimurium is inside macrophages. Moreover, they support the notion of SsrB promoting *S*. Typhimurium virulence, in part, by activating the PhoP protein.

### The SsrB binding site in the *ugtL* promoter is conserved among *S. enterica* serovars that infect warm-blooded animals

The *phoP* gene is required for virulence in *S. enterica* serovars other than Typhimurium that infect a variety of warm-blooded animals. That is, inactivation of the *phoP* gene in *S*. Gallinarum, *S*. Choleraesuis, and *S*. Typhi attenuates virulence in chickens, pigs, and humans, respectively (59–61). Thus, we wondered whether the SsrB activation of *ugtL* is retained in *S. enterica* serovars with different host ranges.

**Figure 6.**
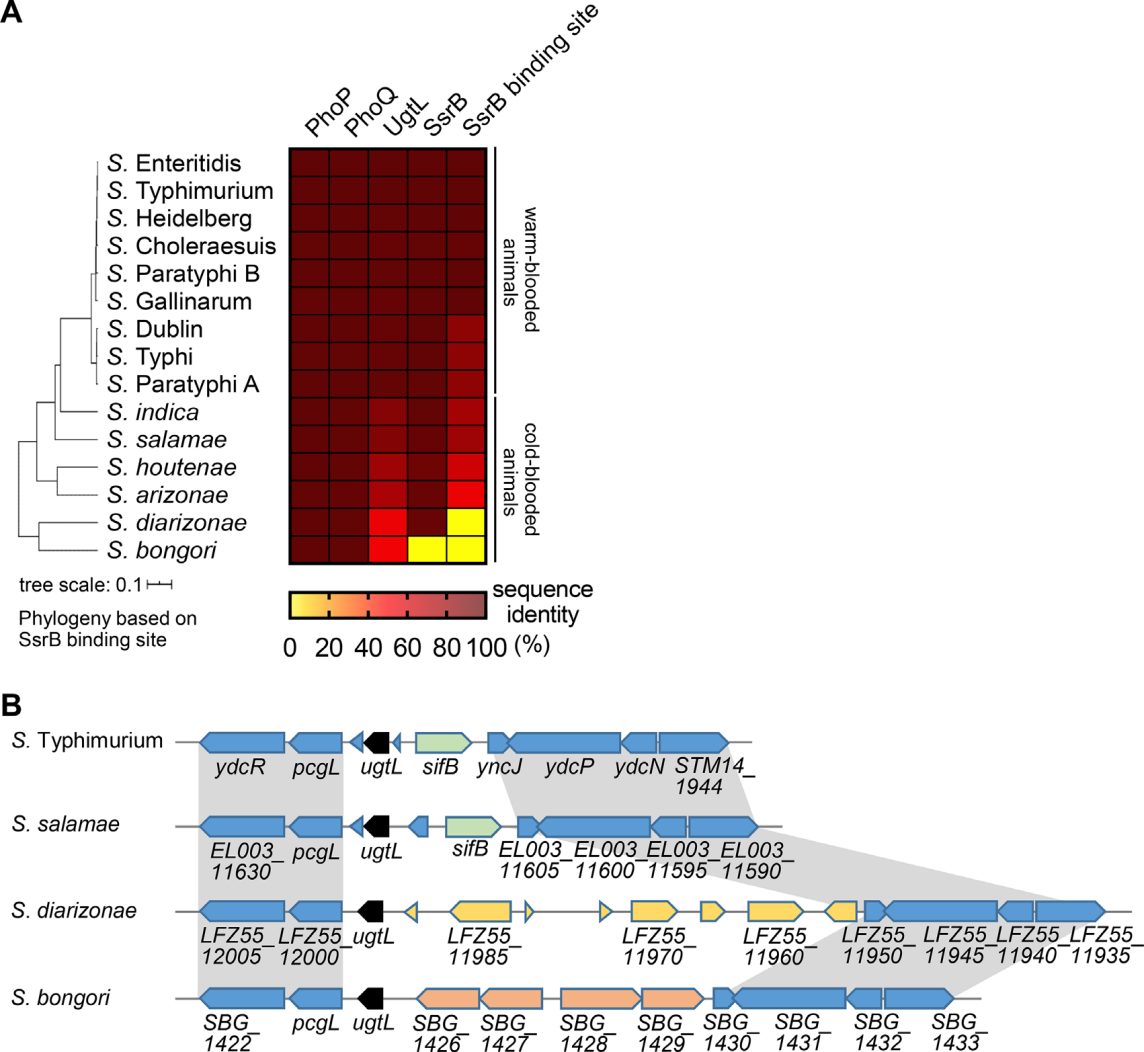
The nucleotide sequences upstream of the *ugtL* gene distinguishes phenotypic properties of *Salmonella* species. (**A**) A heat map of sequence identity among *Salmonella* species and serovars. Sequence identity of the deduced amino acid sequences of the *phoP, phoQ, ugtL and ssrB* genes analyzed using TBLASTN. DNA sequence identity of the SsrB binding region in the *ugtL* promoter (SsrB binding site) was analyzed using BLASTN. Sequences of indicated elements from *S. bongori* (NCTC12419), *S. enterica* subsp. *diarizonae* (SA20044251; *S. diarizonae*), *S. enterica* subsp. *arizonae* (RKS2983; *S. arizonae*), *S. enterica* subsp. *houtenae* (CFSAN000552; *S. houtenae*), *S. enterica* subsp. *salamae* (RSE42; *S. salamae*), *S. enterica* subsp. *indica* (NCTC12420; *S. indica*), *S. enterica* subsp. *enterica* serovar Parayphi A (ATCC11511; *S*. Paratyphi A), *S. enterica* subsp. *enterica* serovar Typhi (Ty2; *S*. Typhi), *S. enterica* subsp. *enterica* serovar Dublin (ATCC39184; *S*. Dublin), *S. enterica* subsp. *enterica* serovar Gallinarum (1984; *S*. Gallinarum), *S. enterica* subsp. *enterica* serovar Paratyphi B (SPB7; *S*. Paratyphi B), *S. enterica* subsp. *enterica* serovar Cholerasuis (SC-B67; *S*. Cholerasuis), *S. enterica* subsp. *enterica* serovar Heidelberg (41578; *S*. Heidelberg), and *S. enterica* subsp. *enterica* serovar Enteritidis (92–0392; *S*. Enteritidis) were compared to those of wild-type *S*. Typhimurium (14028s). % identity values are displayed in color map. The phylogenetic tree was made by the interactive Tree of Life software (v5) based on analysis of the *ugtL* gene and its upstream region containing the SsrB binding site (720 nt upstream from the start codon (AUG) of the *ugtL* gene and whole coding region) using Clustal Omega. (**B**) Schematic of the *ugtL* gene and its neighboring genes in *S*. Typhimurium, *S. salamae*, *S*. *diarizonae*, and *S. bongori*. The conserved regions are indicated with gray color.

The nucleotide sequences corresponding to the *ssrB* coding region and the SsrB binding region in the *ugtL* promoter are highly conserved (>99% and >85%, respectively) among the *Salmonella* serovars that infect warm-blooded animals (Figure 6A). By contrast, the *ugtL* promoter, leader, and coding regions are much less conserved in *ssrB-*containing *S. enterica* subspecies that infect cold-blooded animals. For example, sequences resembling the SsrB binding region are not found in the *ugtL* promoter of *S. enterica* subspecies *diarizonae*, despite this bacterium harboring *ssrB* (Figure 6A and 6B). This and other *Salmonella* subspecies that infect cold-blooded animals have different serovar-specific gene sets in the region occupied by the *sifB* gene in *S*. Typhimurium (Figure 6B). These results suggest that the SsrB binding site in the *ugtL* promoter contributes to the different habitats of individual *S. enterica* serovars.

### Mildly acidic pH activates PhoP in *S. bongori* if *ugtL* is transcribed from a heterologous promoter

**Figure 7.**
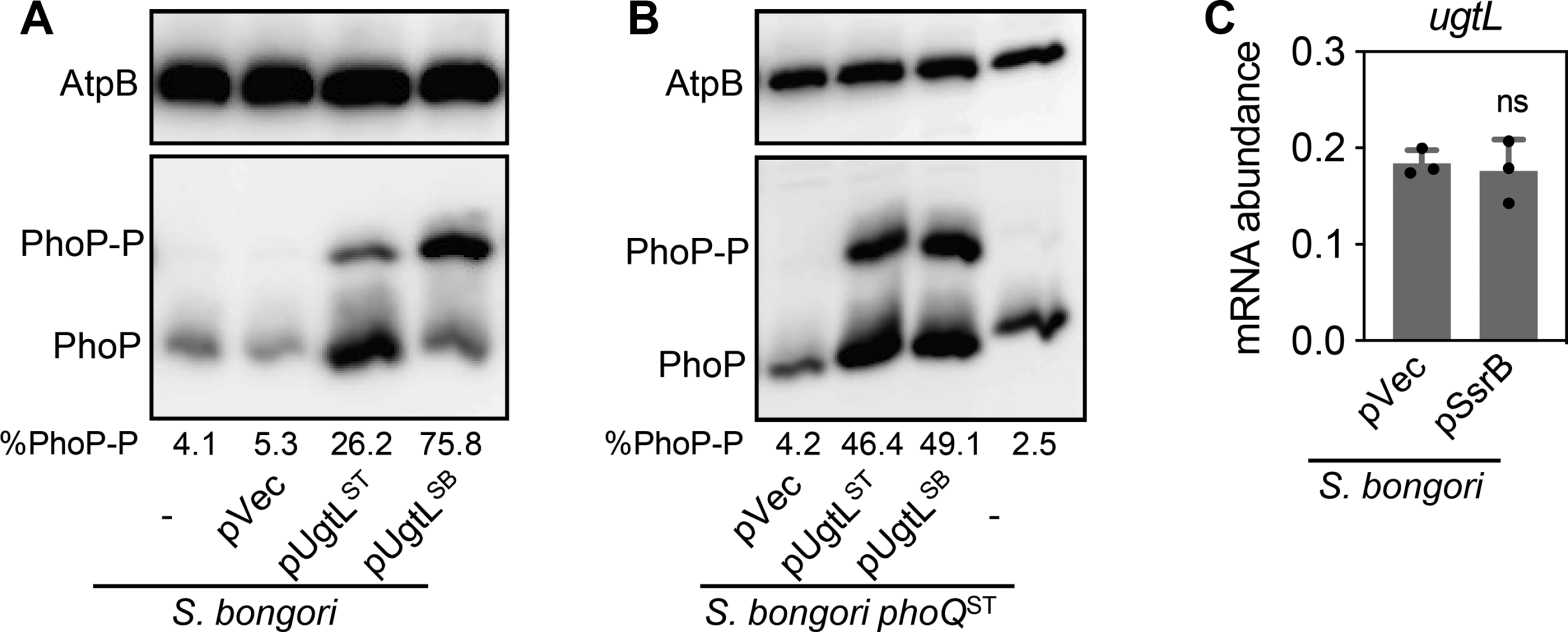
Expression of the *ugtL* gene from a heterologous promoter increases the fraction of PhoP-P in *S. bongori* grown in mildly acidic pH. (A, B) Phos-tag Western blot analysis of crude extracts prepared from wild-type *S. bongori* (**A**) or *S. bongori* harboring *S*. Typhimurium *phoQ* (*phoQ*^ST^) (JC325) (**B**) strains harboring a plasmid expressing the *S*. Typhimurium *ugtL* (pUgtL^ST^) or *S. bongori ugtL* (pUgtL^SB^) gene from a heterologous promoter or an empty vector (pVec), or with no plasmid (–) grown to mid-log phase in N-minimal media with 1 mM of Mg^2+^ at pH 4.9 grown to mid-log phase in N-minimal media with 1 mM of Mg^2+^ at pH 4.9 (acidic pH) with 500 μM of IPTG using antibodies recognizing PhoP or the loading control AtpB. Representatives of at least three independent experiments are shown. Numbers under the blots indicate % phosphorylated PhoP (PhoP-P). (**C**) mRNA abundance of the *ugtL* gene in wild-type *S. bongori* harboring a plasmid expressing the *ssrB* gene (pSsrB) from a heterologous promoter or an empty vector (pVec) grown to mid-log phase in N-minimal media with 1 mM of Mg^2+^ at pH 4.9. The mean and SD from three independent experiments are shown (*n* = 3). Each dot represents individual biological sample. Two-tailed *t*-test with strain with pVec vs. the one with pSsrB; ns, not significant.

The data presented above argue that *S. bongori* fails to activate PhoP/PhoQ in mildly acidic pH because it lacks the *ssrB* gene and the SsrB binding region in the *ugtL* promoter region (Supplemental Figure S6). If this is correct, it should be possible to bypass the requirement for both *ssrB* and the SsrB binding site in the *ugtL* promoter by expressing the *ugtL* gene from a heterologous promoter. As hypothesized, *ugtL-*expressing plasmids increased the PhoP-P-to-PhoP ratio in both wild-type *S. bongori* and in an *S. bongori* derivative expressing the *S*. Typhimurium *phoQ* gene instead of its own (Figure 7A and B). This was true whether the *ugtL* gene originated from *S*. Typhimurium or *S. bongori* (Figure 7A and B). (We note that the *S. bongori* UgtL had higher activity than the *S*. Typhimurium UgtL when investigated in wild-type *S. bongori* (Figure 7A).) By contrast, the vector control had no effect (Figure 7A and B). Furthermore, a plasmid expressing the *ssrB* gene from a heterologous promoter complemented the *ssrB S*. Typhimurium mutant (Figure 2A) but failed to promote *ugtL* transcription in *S. bongori* (Figure 7C) because *S. bongori* lacks the SsrB binding site in the *ugtL-sifB* intergenic region.

## DISCUSSION

The phenotypic properties that distinguish closely related bacterial species are often ascribed to differences in gene content. These differences usually result from horizontal gene transfer, whereby foreign genes confer new abilities upon a recipient organism. Foreign genes, such as those conferring resistance to antibiotics, can operate independently of the ancestral genome, and this is why virtually identical resistance determinants are found in distantly related bacterial species (1,3). By contrast, our results now establish that a horizontally acquired gene can act on the ancestral genome by providing abilities that are realized only by the *simultaneous* presence of horizontally acquired and ancestral genes in the same organism (Figure 1). Therefore, the phenotypic properties that distinguish closely related bacterial species are more accurately ascribed to *both* the intrinsic properties of species-specific genes and their effects on ancestral genes.

### Control of the virulence regulator PhoP by the SsrB protein

**Figure 8.**
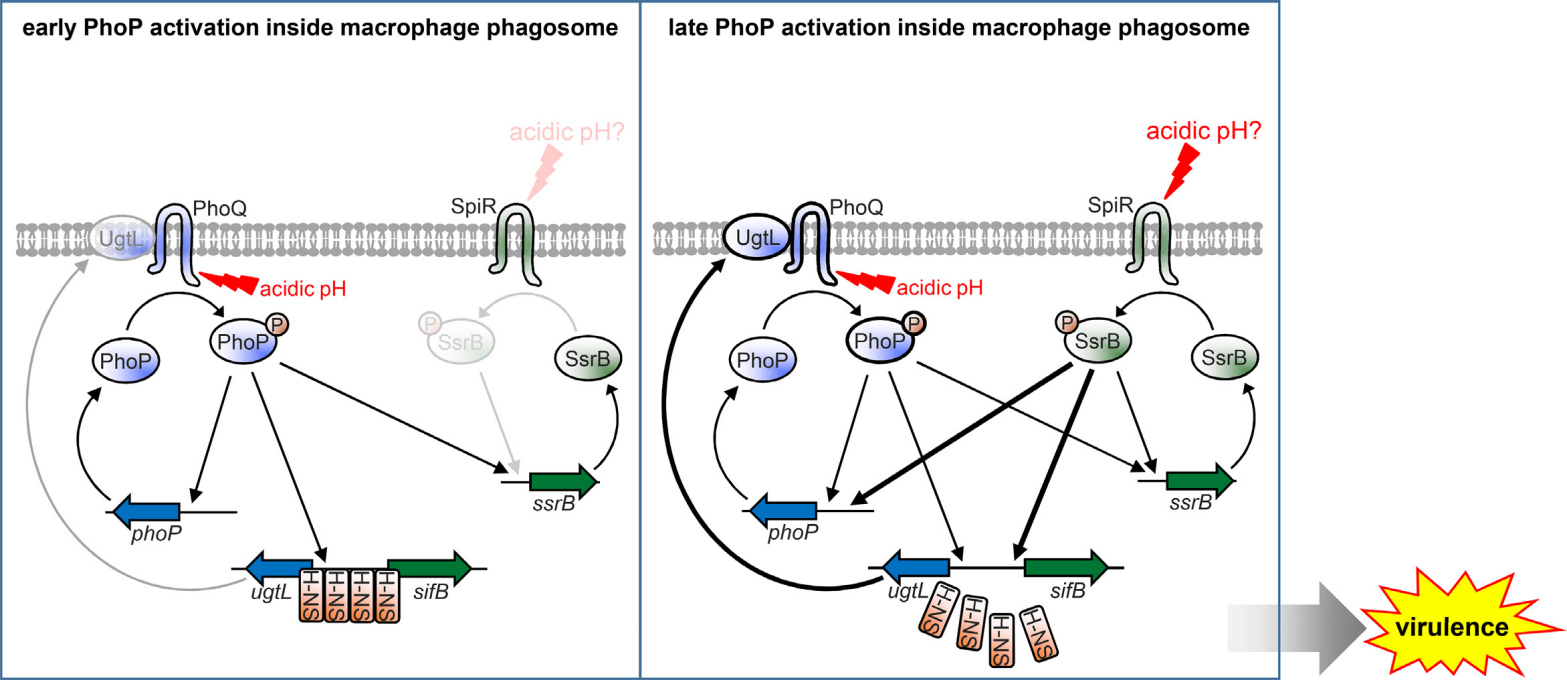
The horizontally acquired *ssrB* gene activates the ancestral regulator PhoP at late times inside macrophages. (Left) At early times following bacterial internalization by macrophages, phagosomal signals, such as mildly acidic pH, stimulate the ancestral sensor PhoQ to promote the phosphorylated state of PhoP. PhoP-P promotes transcription of its target genes, including the *phoP, ugtL*, and *ssrB* genes. The SsrB protein shows little activity at this time. The little *ugtL* expression taking place is not sufficient for full PhoP activation. (Right) At later times following bacterial internalization by macrophages, SpiR activates SsrB in response to signals yet to be identified, thereby promoting transcription of the *ugtL* gene by antagonizing the xenogeneic silencer H-NS. UgtL enhances PhoQ autophosphorylation, further activating PhoP. SsrB also increases *phoP* transcription by directly binding to the *purB* coding region upstream of the *phoP* promoter region. As a consequence, PhoP further increases transcription of its activated genes. The SsrB-mediated PhoP activation is necessary for normal *Salmonella* virulence.

We suggest the following model of how the horizontally acquired *ssrB* gene confers upon *S*. Typhimurium the ability to activate the ancestral PhoP/PhoQ system in mildly acidic pH (Figure 1). The SsrB protein binds to the SsrB binding site in the *ugtL-sifB* intergenic region (Figure 3D), which displaces the foreign gene silencer H-NS and results in *ugtL* transcription (Figure 3EF). The UgtL protein stimulates autophosphorylation of PhoQ (25), which increases the fraction of PhoP-P, thereby promoting transcription of PhoP-activated genes (Figure 3E and Supplemental Figure S7). In addition, SsrB furthers PhoP amounts by increasing *phoP* transcription (Figure 4). In this way, activation of the ancestral PhoP/PhoQ system by the horizontally acquired *ssrB* gene enables *S*. Typhimurium to cause disease (Figures 1 and 8).

The mildly acidic pH of the macrophage phagosome activates the PhoP protein in an *ssrB-*independent manner shortly after bacterial entry (Figure 5B and 8). At 6 h, however, active SsrB promotes *ugtL* expression, thereby enhancing PhoP activation (Figure 5B and 8). Given that the phagosome acidifies less than 1 h after phagocytosis (26,27) and that SsrB shows close to wild-type activity in laboratory media of mildly acidic pH (Supplemental Figure S10), SsrB may be activated by another phagosomal signal(s) at 6 h inside a phagosome.

The DNA binding proteins PhoP, SsrB, and SlyA have been implicated in *ugtL* transcription. PhoP binds at two different sites in the *ugtL* promoter (Supplemental Figure S6) (62) and is necessary to recruit RNA polymerase (63). By contrast, SsrB and SlyA operate as anti-silencers that antagonize silencing by H-NS (Figure 3 and 8) (63). Expression of both SsrB and SlyA is transcriptionally controlled by PhoP (7,64). However, SsrB and SlyA differ in several properties: (i) SsrB is necessary for *ugtL* transcription in mildly acidic pH but not in low Mg^2+^ (Figure 2B, 4E and Supplemental Figure S7), whereas SlyA is necessary for activation in low Mg^2+^ (62,63); (ii) the SlyA binding site is proximal to the transcription start sites of the *ugtL* gene (62), whereas the SsrB binding site is located further upstream (Supplemental Figure S6) and (iii) SlyA is present in both *S. enterica* and *S. bongori*, whereas SsrB is present only in *S. enterica*. Cumulatively, these findings suggest that *S*. Typhimurium employs different DNA binding proteins to aid PhoP transcribe the *ugtL* gene, depending on the identity of the PhoQ inducing signal.

Our findings have implications regarding PhoP/PhoQ-mediated bacterial physiology. First, they raise the possibility of signals altering UgtL abundance impacting PhoP activation status. For example, SsrB responds to redox changes (65). Likewise, the *Salmonella-*specific regulators HilD and SprB (50,66) have been implicated in *ugtL* expression (67). And second, SsrB activation of PhoP is likely to have genome-wide effects, beyond the genes directly controlled by PhoP because: (i) PhoP is a direct transcriptional activator of the *rstA* and *slyA* genes, which specify DNA binding regulatory proteins (63,64,68,69); (ii) PhoP activates the transcriptional regulator PmrA post-translationally (70,71); (iii) PhoP promotes degradation of the gene silencer H-NS (72); and (iv) PhoP reduces proteolysis by different proteases that target pleiotropic regulators (73–75). The PhoP-dependent effects may account, in part, for SsrB controlling expression of ∼5% of the *S*. Typhimurium genome (17,18).

Our findings suggest that the SsrB-mediated induction of the *ugtL* gene taking place inside macrophages (Figure 5B) contributes to virulence in an animal host (Figure 5A and Supplemental Figure S9) by generating active PhoP protein (Figure 5B). Given that PhoP-activated genes, including *ugtL*, are highly induced inside the vacuolar compartment of epithelial cells (76), the PhoP activation mechanisms described in this paper may also take place in such cells during infection, and thus, promote *Salmonella* virulence.

### The genetic basis for phenotypic differences among *Salmonella* species and serovars

The two species that comprise the *Salmonella* genus differ in hundreds of genes (12,13,77). Some of these genes are directly responsible for *S*. Typhimurium being pathogenic and *S. bongori* being largely non-pathogenic. We have now established that this phenotypic difference also results from a *S*. Typhimurium-specific gene acting on genes that are *shared* between the two species (Figure 1). That is, the *S*. Typhimurium-specific *ssrB* gene is necessary for activation of the widespread PhoP protein in mildly acidic pH (Figure 2). *S. bongori* fails to activate PhoP in mildly acidic pH (Figure 7) (29) because it lacks both the *ssrB* gene (12) and the SsrB binding site in the *ugtL* promoter region (Figure 1 and Supplemental Figure S6), and also because of differences in the *phoQ* gene (29). That nucleotide differences in bacterial promoter sequences play a critical role in bacterial evolution is further supported by the dramatic increase in virulence displayed by an African clade of *S*. Typhimurium with a single nucleotide difference in the promoter region of the *pgtE* gene (78).

The PhoP-P-to-PhoP ratio was higher in wild-type *S. bongori* when harboring a plasmid expressing the *S. bongori ugtL* gene than when harboring an isogenic plasmid expressing the *S*. Typhimurium *ugtL* gene (Figure 7A). By contrast, the ratio was similar when the plasmids were present in a *S. bongori* variant expressing the *S. enterica phoQ* gene in place of *S. bongori*’s own *phoQ* gene (Figure 7B). These results suggest co-evolution of the horizontally acquired *ugtL* and the ancestral *phoQ* genes in *Salmonella* species.

A phylogeny based on the ∼2.6-Mb core *Salmonella* sequence (79) resembles that based on the *ugtL* promoter, leader, and coding regions (∼1.2 Kb) (Figure 6A). This analysis suggests that the PhoP activation resulting from the anti-silencing effects of SsrB on the *ugtL* promoter is a key feature of *Salmonella* serovars that infect warm-blooded animals. Moreover, it implies that PhoP activation by mildly acidic pH is dispensable in *Salmonella* serovars that infect cold-blooded animals.

### Different horizontally acquired genes enable *Escherichia coli* to activate PhoP/PhoQ in mildly acidic pH

*E. coli* can activate the PhoP/PhoQ system in mildly acidic pH (80) despite lacking the *ssrB* and *ugtL* genes. This activation requires the regulatory system encoded by the *evgA* and *evgS* genes (81), which have features of horizontally acquired DNA, including an unusually low GC content, absence from closely related species, and being bound by the gene silencer H-NS (82). It is also dependent on EvgA-activated *safA* (83), a horizontally acquired gene that specifies a post-translational activator of the PhoQ protein (84). There is no shared sequence identity between the SafA and UgtL proteins or between the EvgA and SsrB proteins, suggesting that *E. coli* and *S. enterica* independently acquired the ability to activate the ancestral regulatory system PhoP/PhoQ in mildly acidic pH.

## CONCLUDING REMARKS

Finally, the mobile nature of some horizontally acquired genes suggests that, as long as such genes are expressed, they operate regardless of the host genetic background. For example, a gene specifying an antibiotic-inactivating protein typically confers antibiotic resistance to a wide variety of bacterial species. However, our findings present a new paradigm: a horizontally acquired gene regulating an ancestral regulatory system. This paradigm indicates that the activity of conserved ancestral genes can vary across species, depending on which horizontally acquired genes are present in a given organism.

## DATA AVAILABILITY

The data that support the findings of this study are available from the corresponding author on reasonable request.

## Supplementary Material

gkaa813_Supplemental_Files
