# Supplementary material for: Horizontally acquired regulatory gene activates ancestral regulatory system to promote Salmonella virulence
Source: Nucleic Acids Res. 2020 Oct 12;48(19):10832–47. doi: 10.1093/nar/gkaa813 (PMC7641745; doi:10.1093/nar/gkaa813)
Supplement: gkaa813_Supplemental_Files [file gkaa813_supplemental_files.zip › SsrB activates ugtL 58.2 NAR-SD.pdf]

Supplemental Data for

Horizontally acquired regulatory gene promotes *Salmonella* virulence by activating ancestral regulatory system

Jeongjoon Choi[1] and Eduardo A. Groisman[1, 2, *]

[1] *Department of Microbial Pathogenesis, Yale School of Medicine, 295 Congress Avenue, New Haven, CT 06536, USA;* [2] *Yale Microbial Sciences Institute, P.O. Box 27389, West Haven, CT, 06516, USA*

**Correspondence:* eduardo.groisman@yale.edu

***Supplemental Data* contain followings:**

Supplemental Tables S1 and S3.

Supplemental Figures S1 – S10.

References

Supplemental Table S1. Bacterial strains and plasmids used in this study.

Strains or plasmids	Description	Source
***Salmonella enterica* serovar Typhimurium**		
14028s	wild-type	(1)
MS7953s	*phoP*::Tn*10*	(2)
EG9331	*pcgL-MudJ*	(3)
EG10232	*phoP*phoQ*::Tn*10*	(4)
EG11250	*ugtL-lac*	(5)
EG13682	*ugtL*::*kan*	(6)
EG14411	*ssrB*::*cat*	(7)
JC90	*sseA-sseG*::*kan*	This work
JC91	*ssaG-ssaU*::*kan*	This work
JC92	*sseA-ssaU*::*kan*	This work
JC201	*pcgL-MudJ ssrB*::*cat*	This work
JC257	*ugtL-lac ssrB*::*cat*	This work
JC449	*phoP*phoQ*::Tn*10 ssrB*	This work
JC805	*hns-FLAG*	(8)
JC925	*hns-FLAG ugtL*::*kan*	(8)
JC1360	$p_{lac1\text{-}6}$-*ugtL*	This work
JC1373	*ugtL-FLAG*::*kan*	This work
JC1449	$p_{lac1\text{-}6}$-*ugtL ssrB*::*cat*	This work
JC1452	*ugtL*::*kan ssrB*::*cat*	This work
JC1458	attTn*7*:: p_{ycfC}-*purB purB* P*phoP*$_{mu}$	This work
JC1463	attTn*7*:: p_{ycfC}-*purB purB* P*phoP*$_{WT}$ *ssrB*	This work

JC1464	attTn*7*:: p$_{ycfC}$-*purB purB* P*phoP*$_{mu}$ *ssrB*	This work
JC1482	attTn*7*:: p$_{ycfC}$-*purB purB* P*phoP*$_{WT}$	This work
JC1547	*ugtL-sifB*$_{mu}$	This work
JC1548	*ugtL-sifB*$_{mu}$ *ssrB*::*cat*	This work
JC1567	*sifB*::*kan*	This work
JC1582	attTn*7*:: p$_{ycfC}$-*purB purB* P*phoP*$_{WT}$ *ugtL-sifB*$_{mu}$	This work
JC1583	attTn*7*:: p$_{ycfC}$-*purB purB* P*phoP*$_{mu}$ *ugtL-sifB*$_{mu}$	This work
JC1586	attTn*7*:: p$_{ycfC}$-*purB purB* P*phoP*$_{WT}$ *sifB*	This work
JC1625	*hns-FLAG ugtL-sifB*$_{mu}$	This work
JC1723	*ugtL-FLAG*::*kan ssrB*::*cat*	This work
Salmonella bongori		
S3041	wild-type	(9)
JC324	*phoQ::tetAR*	This work
JC325	*phoQ* is replaced by *S. enterica phoQ*	This work
Escherichia coli		
DH5α	F–*sup*E44 Δ*lac*U169 (*f* 80 *lacZ*ΔM15) *hsd*R17 *rec*A1 *end*A1 *gyr*A96 *thi*-1 *rel*A1	(10)

BL21(DE3)	F^- *ompT hsdS gal lon dcm* λ(DE3[*Its857 ind1 Sam7 nin5 lacUV5-T7*])	(11)
**Plasmids**		
pCP20	rep$_{pSC101}^{ts}$ Ap^R Cm^R *FLP*$^+$ *c*I857$^+$	(12)
pKD3	rep$_{R6K□}$ Ap^R FRT Cm^R FRT	(12)
pKD4	rep$_{R6K□}$ Ap^R FRT Km^R FRT	(12)
pKD13	rep$_{R6K□}$ Ap^R FRT Km^R FRT	(12)
pKD46	rep$_{pSC101}^{ts}$ Ap^R p$_{araBAD}$ γ β exo	(12)
pSLC-242	rep$_{R6K□}$ Cm^R p$_{rhaB}$-*relE*	(13)
pH6-SsrBc	rep$_{pMB1}$ Ap^R *lacI*^{q} p$_{lac}$-*ssrBc*	This work
pUHE21-2*lacI*^{q}	rep$_{pMB1}$ Ap^R *lacI*^{q}	(14)
pPhoP	rep$_{pMB1}$ Ap^R *lacI*^{q} p$_{lac}$-*phoP*	(14)
pSsrB	rep$_{pMB1}$ Ap^R *lacI*^{q} p$_{lac}$-*ssrB*	This work
pSsrB^{V197A}	rep$_{pMB1}$ Ap^R *lacI*^{q} p$_{lac}$-*ssrB*(V197A)	This work
pUgtLST	rep$_{pMB1}$ Ap^R *lacI*^{q} p$_{lac}$-*ugtL*ST	(6)
pUgtL^{SB}	rep$_{pMB1}$ Ap^R *lacI*^{q} p$_{lac}$-*ugtL*^{SB}	This work
pFPV25AAV	rep$_{ColE1}$ Ap^R promoterless *gfp-aav*	(15)
pFPV25AAV-P*phoP*	rep$_{ColE1}$ Ap^R p$_{phoP}$-*gfp-aav*	(16)
pFPV25AAV-P*pmrD*	rep$_{ColE1}$ Ap^R p$_{pmrD}$-*gfp-aav*	This work
pFPV25AAV-P*mig-14*	rep$_{ColE1}$ Ap^R p$_{mig-14}$-*gfp-aav*	This work
pGRG36	rep$_{pSC101}^{ts}$ Ap^R p$_{BAD}$-*tnsABCD*	(17)

pGRG-purB	rep_{pSC101}^{ts} Ap^{R} p_{BAD}-*tnsABCD* p_{ycfC}-*purB*	This work

Supplemental Table S2. Oligonucleotides used in this study.

Name	Sequence (from 5′ to 3′)
3203	CCA GCA GCC GCG GTA AT
3204	TTT ACG CCC AGT AAT TCC GAT T
4149	ACC GTG GCA CAA ATG ATG CT
4150	TCG GCA ATC GCC TTA TCT G
4740	TTA GGG ATC CGC CGC TAT CGC ACA GCA C
4803	CGG GAT CCG CCG TAT TGC GTA AAC ACC TC
5900	CGG AAT TCA AGA GTT TGG TCA TCG TCC CGT TG
6044	CGG AAT TCA GTA TCG GAA ACG CTA TCA TAT TC
6627	GGC GAC CGT AGT AAT ATC GAC AA
6628	CTT TCC TCC TGT TCA GCC TGT T
6962	GCA GGA GTA ATA TGT TGG ACA GTC AC
6963	GGG AGA TTG CTG CCC ACC
6964	AAA AGA TTA AAT CGG AGC GGG A
6965	TGA CGC TCC ATC CGC AAT A
7016	ACA TCA TGC TTT TAT GCT TTG GTC
7017	AAA CCA GAA CAA TGG CCT GAA
7225	TTC AGG GTC CAT GTC GCC
7226	CCA CAA AAC TTA TGG ATT TAT GCG T
7295	CAA AGC CGT CAG CTA ATC GTT
7302	TAG GGC AGA AGG CCA ATA CTG AT
13096	GCA TAC GAG CTG ACA TAC TTA TC
13097	CAA CGC TGA TGT AAT TGA GGA ATG

13098	ATG AAT GGT AAT GAT TTG CTC AAC
13099	AAA TGT AGA ATA CTG CTG TAA GGC
13160	CGT TTT GGC GAG CAC TGC CCA GGT TCA AAA TGG CAA GTA AAA TAG CCT GTA GGC TGG AGC TGC TTC G
13161	CCT GAA AAC GAT TAC TCC GGC GCA CGT TGT TCT GGC GTT ACC TGA GCA TTC CGG GGA TCC GTC GAC C
13164	ACG TGC GCC GGA GTA ATC GTT TTC AGG TAT ATA CCG GAT GTT CAT TGT GTA GGC TGG AGC TGC TTC G
13165	AGT GGC CTG AAG AAG CAT ACC AAA AGC ATT TAT GGT GTT TCG GTA GAA TTC CGG GGA TCC GTC GAC C
14217	CTT CAG GTG GTG GCG TAA TAA TGC ATT AT
14224	TCT GTC GAG GAT CCC AAA ATG GGA CAC CAC CAC CAC CAC CAC GCT GAA TTA AAC GCT GAC ACG ACC AAT CA
14225	CAA AAT ATG ACC AAA GCT TAA TAC CAT CGG ACG CCC C
14226	TTT CGC GAG GAT CCC AAA ATG AAA GAA TAT AAG AT
14244	GTC ATG CGC CGC TAT GGT ATT GAA AAA C
14810	GTG ATT ACC ACT GTG CGC GGG CAA GGA TAC CTT TTC GAA TTG CGC TAA TGA ATA AAT TTG CTC GCT TAA GAC CCA CTT TCA CAT T
14811	GTA TCC GCA GGC TGG TAT CTG ACA CCG ATT ATA ACG GAT GCT TAG CGA GAT GCG CGG CAG AAC GCC CCT AAG CAC TTG TCT CCT G
14821	GCT GAA CTG CGG GAA AGC CAT ACC ATT GAT GTT CTC ATG GGG CGC CTG CGA AAA AAA ATA CAG GCC CAG TAT CCG CAT GAT GTG ATT ACC ACT GT

14822	GTT TTT GCC AGT GAC GTT CAA GAA AGT CGG GCC AGT TAA GAG TTA ATT GGT ATT CCA TGT TAA GTA TCC GCA GGC TGG TAT CTG ACA CCG ATT AT
14950	GTG TTG ATG CCG AAG GAA TGA AAC AGT TTA TTG ATA GTC TGG CCC TGC CGG AAG TTA AGA CCC ACT TTC ACA TT
14951	CAT TAT TTA AGT TCG TCG ACC AGA GTC ACA GCG CGA CCG ATA TAA TTT GCC GGC GTC ACC CTA AGC ACT TGT CTC CTG
14952	GTG TTG ATG CCG AAG GAA TGA AAC AGT TTA TTG ATA GTC TGG CCC TGC CGG AAG CAG AAA AAA GCC ATC TTA AAC GCG CGA CGC CGG CAA ATT ATA TCG GTC GCG CTG TGA CTC TGG TCG ACG AAC TTA AAT AAT G
14953	CAT TAT TTA AGT TCG TCG ACC AGA GTC ACA GCG CGA CCG ATA TAA TTT GCC GGC GTC GCG CGT TTA AGA TGG CTT TTT TCT GCT TCC GGC AGG GCC AGA CTA TCA ATA AAC TGT TTC ATT CCT TCG GCA TCA ACA C
15755	GCT ACA GGT TCA TAA AGC GAC AGA GTT ACT TAA C
15756	GTT AAG TAA CTC TGT CGC TTT ATG AAC CTG TAG C
16058	CGG GAT CCA GGA GGC TCA AAA TGA GGA AAT CAG ATC GCT ATA CGC ATG GG
16059	ACG TAA GCT TAT TAT GAC CCA TAT CTT CTG GTC AAC TGG TAG G
16540	CCT AGT AAG CCA CGT TTT AAT TAA TCA GAT CCG CCC AGG GGC ATG AGC ACC CAC GCC TGA T
16541	AAT TTG TTT TGC CTG AGT CGT CAG GCG ATG GTA TCA AAT AAA ATA CAC TTC ATC TTT TAA

16542	CAT CGC CTG ACG ACT CAG GCA AAA CAA ATT
16543	GTC GAC GCG GCC GTG GCG CGC CTC CTA GGT GCA GGC AGG CAT TAT TTA AGT TCG TCG ACC AG
16655	ATT CTC ACA TCA TCA TGT ACT ATG AGT AAT GAT TAA TTA CGC ACT ATA TTA TTT TTA GAG GTG TAG GCT GGA GCT GCT TCG AAG TTC CTA TAC TTT CTA G
16658	CAG GAT GCT GTC TTT TCG TGA ATT TCA CCA TCT GAT TTC TTC ATT TTG AGC CTC CTT CCA CAC AAC ATA AAC ATA AAA AGC TTA AAG TGT AAA GAT ATG AAT ATC CTC CTT AGT TCC TAT TCC GAA GTT CCT AT
16686	GGT TCA GCG CTT GTC CAA AAA ATT TGG GGC ACA GGA TGT TTC TTC ACG CCC GGA CTA CAA GGA CGA CGA TGA CAA GTG ATG TAG GCT GGA GCT GCT TCG
16687	AGA TAA TTC GGT CTG CTG GTC ATA GCC ATT ATT CAG TAA GAC CGC AGG TTG CAG CGG CGG AAT ATG AAT ATC CTC CTT AGT TC
16869	CGT GCA GCC GTG CTC TGG CAT CAG GTA GGC GGT GGT CGC CTA CAG CTT ATT GTA GGC TGG AGC TGC TTC G
16870	TCG TCA AGC AGA TGA TCG CGG TTT ACT TCC AGC TTG CTG ACG CCC TTC AGA TAT GAA TAT CCT CCT TAG TTC
16879	ATG GAA CTC AGA AGA ATA TCT GTA AAT
16880	CCA TTT GGT CCG GTA ATA ATA ATT GT
17220	CTG GAA TAG GTG GTA TTC GAA ATA TTA TCC CAT GTT GCC CAT CGG TTT GCC TAT CTG TAG GCT GGA GCT GCT TCG

17221	GTT TTA TCT GAC ATA TTT CAT GGC CAG GAG GCG TGG GCA TGA CTA AAG CTA CGG GAT ATG AAT ATC CTC CTT AGT TC
17222	AAT CCT CTC CCG ATA GTA ATT GGC A
17223	AAT ATA GTG CGT AAT TAA TCA TTA CTC ATA GT
17234	AGG TGG TAT TCG AAA TAT TAT CCC ATG TTG CCC ATC GGT TTG CCT ATC GAC CCG TAG CTT TAG TCA TGC CCA CGC CTC CTG GCC ATG AAA TAT GTC AG
17235	CTG ACA TAT TTC ATG GCC AGG AGG CGT GGG CAT GAC TAA AGC TAC GGG TCG ATA GGC AAA CCG ATG GGC AAC ATG GGA TAA TAT TTC GAA TAC CAC CT
17529	TAC GTT ACT ATG GGA AAA GAT TAA AGA TTT
17530	AGA GGC AAC ATC ACA TAA TTC CTT TAT

Supplemental Table S3. Source data for β-galactosidase assays and fluorescence assays. The source data for β-galactosidase assays and GFP assays are provided in a separate excel file.

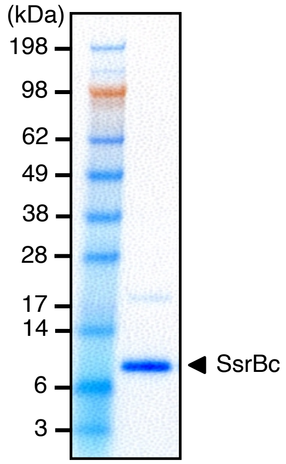

Supplemental Figure S1. Analysis of purified SsrBc protein, related to Figure 3 and 4.

Purified SsrBc was resolved in 4-12% NuPAGE gels and stained using coomassie blue.

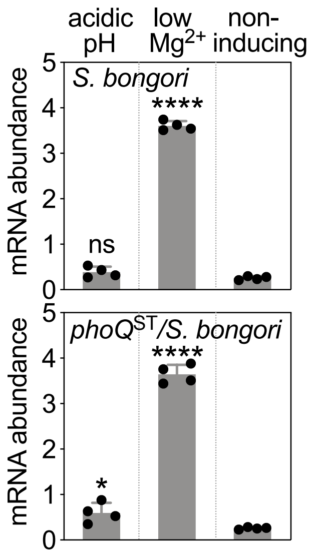

Supplemental Figure S2. ***S. bongori*** **fails to promote** ***phoP*** **transcription in mildly acidic pH even when harboring the** ***phoQ*** **gene from** ***S. enterica*****, related to Figure 2.** mRNA abundance of the *phoP* gene produced by wild-type *S. bongori* (S3041) and an isogenic derivative harboring the *S.* Typhimurium *phoQ* gene instead of its own (*phoQ*ST) (JC325) grown to mid-log phase in N-minimal media with 1 mM of Mg^{2+} at pH 4.9 (acidic pH), 10 μM Mg^{2+} at pH 7.6 (low Mg^{2+}), or 1mM Mg^{2+} at pH 7.6 (non-inducing). The mean and SD from four independent experiments are shown (n=4). Each dot represents individual biological sample. Two-tailed *t*-test with non-inducing vs. indicated condition; ns, not significant, *$P < 0.05$, ****$P < 0.0001$.

```
S.Typhimurium_14028  MKKSDGEIHEKTASWGILQSEWLRKCGRLLLLLLYRFVIGWAFFQLLAMIVAGIFLLGIL 60
S.bongori_NCTC12419  MRKSDRYTHGKRPLWYVLESDLLNNPLRFLLLLVWRFVIGWLLFQLVSIIVLGTLLLGIL 60
                     *:***   * *   * :*:*: *.:  *:****::****** :***:::** * :*****

S.Typhimurium_14028  LFHPIIFVQTIAITEKLNHASLDLWHILKLCLWHYGIIAGFIFMAECTLSKSIRQVQRLS 120
S.bongori_NCTC12419  LFYPAAVVQAIATTEKLNHVSIELWHTLKLCTWHYGVIAGFVFMLGCTLNKGLKQAYQLT 120
                     **:*  .**:** ******.*::*** **** ****:****:**  ***.*.::*. :*:

S.Typhimurium_14028  KKFGAQDVSSRP 132
S.bongori_NCTC12419  RRYGS------- 125
                     :::*:
```

Supplemental Figure S3. Alignment of the deduced amino acid sequences of the *ugtL* genes from *S.* Typhimurium and *S. bongori*, related to Figure 2. Deduced amino acid sequence of the *ugtL* gene from *S.* Typhimurium (*S.* Typhimurium) 14028s aligned with that of *S. bongori* NCTC12419 using Clustal Omega. The asterisk indicates identity, colon indicates conservative substitution, and period indicates semi-conservative substitution.

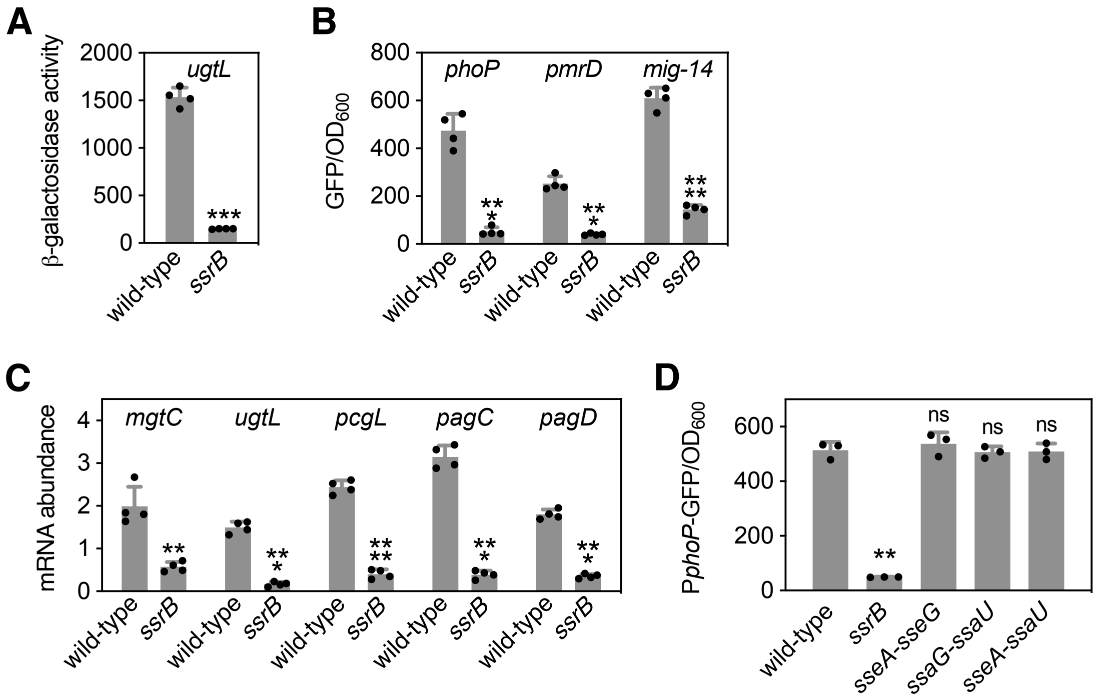

Supplemental Figure S4. SsrB promotes expression of PhoP-activated genes in mildly acidic pH independently of SPI-2 genes, related to Figure 2. (**A**) β-galactosidase activity produced from a chromosomal *ugtL-lacZ* fusion in wild-type (EG11250) and *ssrB* (JC257) *S.* Typhimurium grown to mid-log phase in N-minimal media with 1 mM of Mg^{2+} at pH 4.9. (**B, D**) Fluorescence produced from (**B**) P*phoP-gfp*, P*pmrD-gfp*, and P*mig-14-gfp* transcriptional fusions displayed by wild-type (14028s) and *ssrB* (EG14411) *S.* Typhimurium or (**D**) a P*phoP-gfp* transcriptional fusion displayed by wild-type (14028s), *ssrB* (EG14411), *sseA-sseG* (JC90), *ssaG-ssaU* (JC91), and *sseA-ssaU* (JC92) *S.* Typhimurium grown to mid-log phase in N-minimal media with 1 mM of Mg^{2+} at pH 4.9. (**C**) mRNA abundance of the *mgtC, ugtL, pcgL, pagC* and *pagD* genes produced by wild-type (14028s) and *ssrB* (EG14411) *S.* Typhimurium grown to mid-log phase in N-minimal media with 1 mM of Mg^{2+} at pH 4.9. The mean and SD from at least three independent experiments are shown (**A**, **B**, **D**, n=4; **C**, n=3). Each dot represents individual biological sample. Two-tailed *t*-test with the *ssrB* mutant vs. wild-type (**A**-

C). One-way ANOVA with Brown-Forsythe and Welch tests (wild-type vs. others) (**D**). ns, not significant; **$P < 0.01$, ***$P < 0.001$, ****$P < 0.0001$.

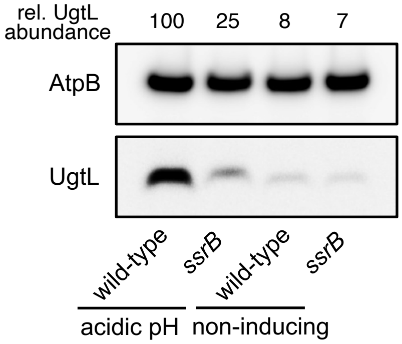

Supplemental Figure S5. SsrB is required for wild-type UgtL protein abundance during grown in mildly acidic pH, related to Figure 3. Western blot analysis of crude extracts prepared from wild-type (JC1373) and *ssrB* (JC1723) *S.* Typhimurium strains with *ugtL-FLAG* at the normal chromosomal location grown to mid-log phase in N-minimal media with 1 mM of Mg^{2+} at pH 4.9 (acidic pH) or 1mM Mg^{2+} at pH 7.6 (non-inducing) using antibodies recognizing the FLAG epitope or the loading control AtpB. Representatives of at least three independent experiments is shown. Numbers above the blot indicates relative amounts of UgtL protein normalized to wild-type grown in acidic pH.

```
S.Typhimurium_14028s       ------------------GAGAGATAGCACTTTGAGAAAACATCTCACTCTTTAAAAATC     42
S.bongori_NCTC12419        TTAGAGATAATAACAGTTGCGGAATCTCATGTTGAGAAATCACCATCAGTATATA-----     55
                                             * *  **  **  ******** ** *       *  *

S.Typhimurium_14028s       CTCTCCCGATAGTAATTGGCATAATGTAGACCACAAGTGATTATATGATACTTCATTACT     102
S.bongori_NCTC12419        -----CAGATGAGTAAAGCGATAGAGT-------GGCTGAGTCTATTATGA----ATAGT     99
                                * ***    *  *  ***  **          *** * *** **       ** *

S.Typhimurium_14028s       GGAATAGGTGGTATTCGAAATATTATCCCATGTTGCCCATCGGTTTGCCTATCGGTGAAA     162
S.bongori_NCTC12419        GTCATGA-TACCACTTAAGAACATATCTGGTATTGATCAGGAATCTAACTCACAGCAGAA     158
                           *  **   *   * *  * *   ****   * ***  **    * *  **  * *   **
                                     DNA region containing SsrB binding site
S.Typhimurium_14028s       CACCTGATTTTTGCTTTGTCCTGAACCGTCAACATTATTGTTCAATTGTTCAAATCGACC     222
S.bongori_NCTC12419        T--------GCTTATTTTAGCAAAATTAAGGGATTAATAGTAAAATTCTCAGACGTGGCG     210
                                      *  ***   *  **         * ** **  **** *   *   * *

S.Typhimurium_14028s       CGTAGCTTTAGTCATGCCCACGCCTCC----TGGCCATGAAATATGTCAGATAAAACGAA     278
S.bongori_NCTC12419        AAACGTTTCGCTATTAGTTAAATCTCATTTAACACTTTGGTTTATATTATATTTCCTGAT     270
                               * **   *  *    *   ***        *  **   *** * * **     **

S.Typhimurium_14028s       TGAAAGTAAAA-----CGGTTTT------CTTAATTCTCACATCATCATGTACTATGAGT     327
S.bongori_NCTC12419        TATAAGTAACAACTCACTGCTTAGTACGCTCACATTCGAACCTTTTCTTATAATTACTAT     330
                           *  ****** *     * * **           ****  ** *  ** * ** *     *
                                                                  PhoP binding site
S.Typhimurium_14028s       AATGATTAATTACGCACTATATTATTTTTAGAGAAAGTAAATAGTTGCTC-AACCGTGTA     386
S.bongori_NCTC12419        ATGCATTAATTCTCGTCAGCATTATTTTTTAAAAAAGTAAACTTTTACTCAACCAATATA     390
                           *   *******     *   *********  * ********   ** *** * *  * **
                                              PhoP binding site
S.Typhimurium_14028s       GAAATTGTCTTATAAGAAGTTAAACTAAAAGTATTATTAGGCTAACAACAATGAGATGTT     446
S.bongori_NCTC12419        AATATTGTGCTACAAAAAATCATGCTGAATGTATTATTAGATTAACAAGGATGTGATGTC     450
                            * *****  ** ** ** * *  ** ** **********  ******  *** *****

S.Typhimurium_14028s       TAGCGGTAGGGCAGAAGGCCAATACTGATAGTG--CTTATGATATAAATATCTTACTCTT     504
S.bongori_NCTC12419        TTAAGCTGTGCAGGCATGGTACCAACTCCGGCAGGGGTATGAAAAATATTTTTTCGCTAT     510
                           *   * *  *   * * *  *  *      *      ***** * * ** * **     *

S.Typhimurium_14028s       TAGTTTTTGTCTTAATTATATTTGTTGTAACGATTAGCTGACGGCTTTGTTTCCAGTTGG     564
S.bongori_NCTC12419        AAAA----AACTACAATGCTTCTGATGGTAATTATTGCTG--ACTGCATTAATCAGTTAG     564
                            *        **  * *   * ** **  *    * ****         *   ***** *

S.Typhimurium_14028s       GCGATAAAATTATAAAAACCTGCGAGGAGGCTCAAAATGAAGAAATCAGATGGTGAAATT     624
S.bongori_NCTC12419        TTGATAATAATATTAAAACCTGTGAGGTATCTAATTATGAGGAAATCAGATCGCTATACG     624
                             ***** * *** ******** ****   ** *  **** ********** *  * *

S.Typhimurium_14028s       CACGAAAAGACAGCATCCTGGGGCATTTTGCAGTCAGAATGGCTAAGGAAATGTGGACGG     684
S.bongori_NCTC12419        CATGGGAAAAGGCCACTTTGGTATGTGCTGGAATCGGATTTGCTAAACAACCCGTTGCGA     684
                           ** *  ** *   **   ***    *  ** * ** ** * *****  **       **

S.Typhimurium_14028s       CTATTATTGCTGTTACTTTACCGTTTCGTTATCGGA                  720
S.bongori_NCTC12419        TTTTTATTGTTGTTAGTATGGCGCTTTGTGATCGGA                  720
                            * ****** ***** * *  ** ** ** ******
```

Supplemental Figure S6. The SsrB binding site in the *ugtL* promoter region of *S* Typhimurium is not conserved in *S. bongori*, related to Figure 3. DNA region upstream of and part of the coding region of the *ugtL* gene of *S* Typhimurium strain 14028s aligned with that of *S. bongori* NCTC12419 using Clustal Omega. The asterisk indicates identity; the green box indicates the DNA region containing the SsrB binding

site; the blue boxes indicate the PhoP binding sites; the red boxes indicate start codon of the *sifB* (upstream one) and the *ugtL* (downstream one) genes; two bold red nucleotides 'A' indicate the transcription start sites of the *ugtL* gene.

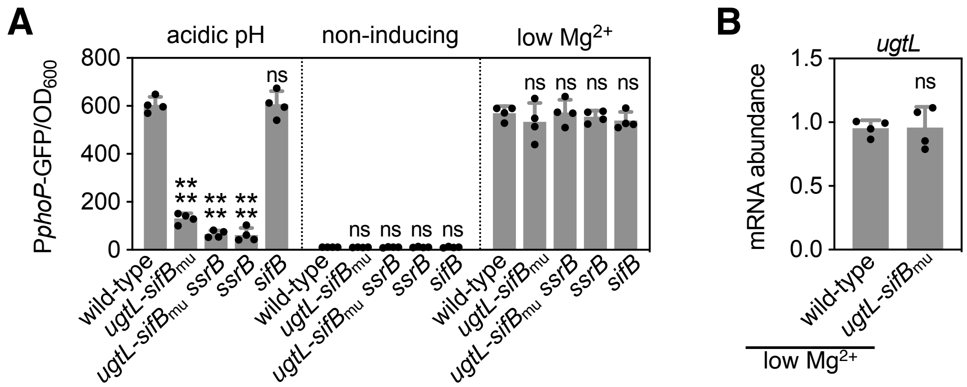

Supplemental Figure S7. SsrB is necessary for PhoP activation in mildly acidic pH but not for transcription of the *ugtL* gene in low Mg^{2+}, related to Figure 3. (**A**) Fluorescence produced from a P*phoP-gfp* transcriptional fusion displayed by wild-type (14028s), *ugtL-sifB*$_{mu}$ (JC1547), *ugtL-sifB*$_{mu}$ *ssrB* (JC1548), *ssrB* (EG14411), *sifB* (JC1567) *S.* Typhimurium. Bacteria were grown to mid-log phase in N-minimal media with 1 mM of Mg^{2+} at pH 4.9 (acidic pH), 10 μM Mg^{2+} at pH 7.6 (low Mg^{2+}), or 1mM Mg^{2+} at pH 7.6 (non-inducing). (**B**) mRNA abundance of the *ugtL* gene produced by wild-type (14028s) and *ugtL-sifB*$_{mu}$ (JC1547) *S.* Typhimurium grown to mid-log phase in N-minimal media with 10 μM of Mg^{2+} at pH 7.6. The mean and SD from four independent experiments are shown (n=4). Each dot represents individual biological sample. One-way ANOVA with Brown-Forsythe and Welch tests (wild-type vs. others) (**A**). Two-tailed *t*-test with wild-type vs. *ugtL-sifB*$_{mu}$ (**B**). ns, not significant, ****$P < 0.0001$.

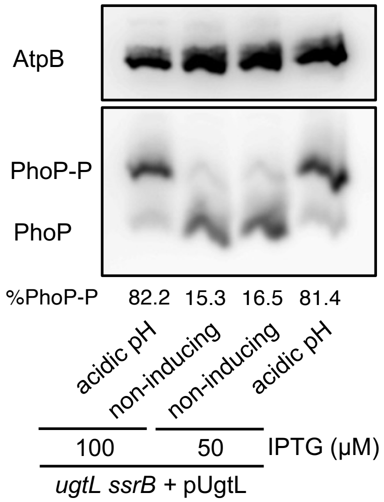

Supplemental Figure S8. A mildly acidic pH is necessary for PhoP activation even when UgtL is expressed from a heterologous promoter, related to Figure 3. Phos-tag Western blot analysis of crude extracts prepared from *ugtL ssrB* (JC1723) *S.* Typhimurium strains with a plasmid expressing *S.* Typhimurium *ugtL* grown to mid-log phase in N-minimal media with 1 mM of Mg^{2+} at pH 4.9 (acidic pH) or at pH 7.6 (non-inducing) supplemented with indicated concentrations of IPTG using antibodies recognizing PhoP or the loading control AtpB. A representative of at least three independent experiments is shown. Numbers under the blots indicate % phosphorylated PhoP (PhoP-P).

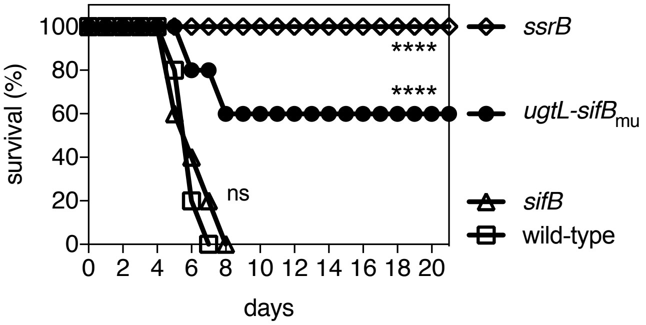

Supplemental Figure S9. SsrB activation of *ugtL* transcription is necessary for *S.* Typhimurium virulence in mice, related to Figure 5. Survival of C3H/HeN mice inoculated intraperitoneally with ~2×10^4 wild-type (14028s), *ugtL-sifB*$_{mu}$ (JC1547), *sifB* (JC1567), and *ssrB* (EG14411) *S.* Typhimurium. Data are representatives of two independent experiments, which produced similar results, n=5 mice per each experimental group. Mantel-Cox test was performed between wild-type and isogenic mutant *Salmonella* infected mice; ns, not significant, ****$P < 0.0001$.

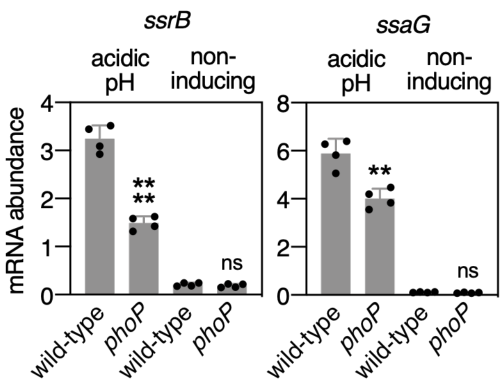

Supplemental Figure S10. SsrB activates the *ssaG* gene in the absence of PhoP under mildly acidic pH, related to Figure 3 and 5. mRNA abundance of the *ssrB* and *ssaG* genes produced by wild-type (14028s) and *phoP* (MS7953s) *S*. Typhimurium grown to mid-log phase in N-minimal media with 1 mM of Mg^{2+} at pH 4.9 or 7.6 (acidic pH or non-inducing). The mean and SD from four independent experiments are shown (n=4). Each dot represents individual biological sample. Two-tailed *t*-test with wild-type vs. *phoP*. ****$P < 0.0001$.

Supplemental References

1. Fields, P.I., Swanson, R.V., Haidaris, C.G. and Heffron, F. (1986) Mutants of *Salmonella* Typhimurium that cannot survive within the macrophage are avirulent. *Proc Natl Acad Sci U S A*, **83**, 5189-5193.
2. Fields, P.I., Groisman, E.A. and Heffron, F. (1989) A *Salmonella* locus that controls resistance to microbicidal proteins from phagocytic cells. *Science*, **243**, 1059-1062.
3. Soncini, F.C., Garcia Vescovi, E., Solomon, F. and Groisman, E.A. (1996) Molecular basis of the magnesium deprivation response in *Salmonella* Typhimurium: identification of PhoP-regulated genes. *Journal of bacteriology*, **178**, 5092-5099.
4. Chamnongpol, S. and Groisman, E.A. (2000) Acetyl phosphate-dependent activation of a mutant PhoP response regulator that functions independently of its cognate sensor kinase. *Journal of molecular biology*, **300**, 291-305.
5. Hilbert, F., Garcia-del Portillo, F. and Groisman, E.A. (1999) A periplasmic D-alanyl-D-alanine dipeptidase in the gram-negative bacterium *Salmonella enterica*. *Journal of bacteriology*, **181**, 2158-2165.
6. Shi, Y., Cromie, M.J., Hsu, F.F., Turk, J. and Groisman, E.A. (2004) PhoP-regulated *Salmonella* resistance to the antimicrobial peptides magainin 2 and polymyxin B. *Molecular microbiology*, **53**, 229-241.
7. Bijlsma, J.J. and Groisman, E.A. (2005) The PhoP/PhoQ system controls the intramacrophage type three secretion system of *Salmonella enterica*. *Molecular microbiology*, **57**, 85-96.
8. Choi, J. and Groisman, E.A. (2017) Activation of master virulence regulator PhoP in acidic pH requires the *Salmonella*-specific protein UgtL. *Sci Signal*, **10**, eaan6284.

9. Boyd, E.F., Wang, F.S., Whittam, T.S. and Selander, R.K. (1996) Molecular genetic relationships of the salmonellae. *Appl Environ Microbiol*, **62**, 804-808.

10. Hanahan, D. (1983) Studies on transformation of *Escherichia coli* with plasmids. *Journal of molecular biology*, **166**, 557-580.

11. Studier, F.W. and Moffatt, B.A. (1986) Use of bacteriophage T7 RNA polymerase to direct selective high-level expression of cloned genes. *Journal of molecular biology*, **189**, 113-130.

12. Datsenko, K.A. and Wanner, B.L. (2000) One-step inactivation of chromosomal genes in *Escherichia coli* K-12 using PCR products. *Proc Natl Acad Sci U S A*, **97**, 6640-6645.

13. Khetrapal, V., Mehershahi, K., Rafee, S., Chen, S., Lim, C.L. and Chen, S.L. (2015) A set of powerful negative selection systems for unmodified Enterobacteriaceae. *Nucleic Acids Res*, **43**, e83.

14. Soncini, F.C., Vescovi, E.G. and Groisman, E.A. (1995) Transcriptional autoregulation of the *Salmonella* Typhimurium *phoPQ* operon. *Journal of bacteriology*, **177**, 4364-4371.

15. Choi, J. and Groisman, E.A. (2013) The lipopolysaccharide modification regulator PmrA limits *Salmonella* virulence by repressing the type three-secretion system Spi/Ssa. *Proc Natl Acad Sci U S A*, **110**, 9499-9504.

16. Choi, J. and Groisman, E.A. (2016) Acidic pH sensing in the bacterial cytoplasm is required for *Salmonella* virulence. *Molecular microbiology*, **101**, 1024-1038.

17. McKenzie, G.J. and Craig, N.L. (2006) Fast, easy and efficient: site-specific insertion of transgenes into enterobacterial chromosomes using Tn*7* without need for selection of the insertion event. *BMC Microbiol*, **6**, 39.
